# Assessing effects of the entomopathogenic fungus *Metarhizium brunneum* on soil microbial communities in *Agriotes* spp. biological pest control

**DOI:** 10.1093/femsec/fix117

**Published:** 2017-09-11

**Authors:** Johanna Mayerhofer, Sonja Eckard, Martin Hartmann, Giselher Grabenweger, Franco Widmer, Adrian Leuchtmann, Jürg Enkerli

**Affiliations:** 1 Molecular Ecology, Agroscope, CH-8046 Zurich, Switzerland; 2 Ecology of Noxious and Beneficial Organisms, Agroscope, CH-8046 Zurich, Switzerland; 3 Forest Soils and Biogeochemistry, Swiss Federal Research Institute WSL, CH-8903 Birmensdorf, Switzerland; 4 Plant Ecological Genetics, Institute of Integrative Biology, ETH Zurich, CH-8092 Zurich, Switzerland

**Keywords:** inundative release, biological control agent, amplicon sequencing, non-target effect, fungal inoculant, next-generation sequencing

## Abstract

The release of large quantities of microorganisms to soil for purposes such as pest control or plant growth promotion may affect the indigenous soil microbial communities. In our study, we investigated potential effects of *Metarhizium brunneum* ART2825 on soil fungi and prokaryota in bulk soil using high-throughput sequencing of ribosomal markers. Different formulations of this strain, and combinations of the fungus with garlic as efficacy-enhancing agent, were tested over 4 months in a pot and a field experiment carried out for biological control of *Agriotes* spp. in potatoes. A biocontrol effect was observed only in the pot experiment, i.e. the application of FCBK resulted in 77% efficacy. Colony counts combined with genotyping and marker sequence abundance confirmed the successful establishment of the applied strain. Only the formulated applied strain caused small shifts in fungal communities in the pot experiment. Treatment effects were in the same range as the effects caused by barley kernels, the carrier of the FCBK formulation and temporal effects. Garlic treatments and time affected prokaryotic communities. In the field experiment, only spatial differences affected fungal and prokaryotic communities. Our findings suggest that *M. brunneum* may not adversely affect soil microbial communities.

## Teaser

Formulated *Metarhizium brunneum* has small and temporal effects on fungal communities in a pot experiment and no effects were detected in the field.

## INTRODUCTION

Soil is a complex and dynamic environment providing habitats for a tremendous number and diversity of soil microorganisms (Nannipieri *et al.*^2003^). It has been estimated that 1 g of soil may harbor up to 10^10^ bacterial and 10^6^ fungal cells and thousands of bacterial and fungal species (Torsvik, Goksoyr and Daae 1990; Bridge and Spooner 2001; Roesch *et al.*^2007^; Trevors 2009). Soil microorganisms provide a wealth of functions. They play a central role in nutrient cycling and the formation and maintenance of soil structure, they contribute to plant health and they are involved in the natural regulation of insects, pathogens and weeds (Kennedy 1999). All together these functions are vital for maintaining productivity in agriculture and it is important to understand which abiotic and biotic factors, including agricultural practices, may adversely affect microbial communities. The potential impacts of a number of factors including time, space and climate on microbial communities have been investigated in various systems (Lauber *et al.*^2013^; Tedersoo *et al.*^2014^; O’Brien *et al.*^2016^). Likewise, the effects of edaphic factors or anthropogenic activities, such as land use, soil compaction and pesticide applications have been studied (Lauber *et al.*^2013^; Hartmann *et al.*^2014^; Jacobsen and Hjelmsø 2014).

The ability of microorganisms to regulate insects, pathogens and weeds has been recognized as an important function with potential use in agriculture more than a century ago (Krassilstschik 1888; Prior 1996; Zimmermann 2007). Since then a variety of microorganisms has been identified and commercialized as microbial pesticides also known as biological control agents (BCA; Faria and Wraight 2007; Lugtenberg 2015). Microbial control usually implies application of large amounts of infective propagules of a BCA to soils under treatment. For instance, about 10^12^–10^14^ propagules of entomopathogenic fungi are applied per hectare translating into 10^5^ conidia per cm^2^ of soil (Jaronski 2010). Such high loads of propagules may have unintended side effects leading to changes in soil microbial community structures. The European Union therefore has included an assessment of potential effects on indigenous soil microorganisms in the registration process of biological pesticides (Commission regulation No. 544/2011). Most studies assessing the effects of applied microorganisms on soil microbial communities have revealed only small or transient effects (Trabelsi and Mhamdi 2013; Kröber *et al.*^2014^; Zimmermann *et al.*^2016^), but little is known about potential effects of the application of entomopathogenic fungi (Hu and St Leger 2002; Rai and Singh 2002; Kirchmair *et al.*^2008^; Schwarzenbach, Enkerli and Widmer 2009; Hirsch *et al.*^2013^).

*Agriotes* spp. Eschscholtz (Elateridae) are major soil dwelling pests in the Holarctic (Kudryavtsev *et al.*^1993^; Vernon, Lagasa and Philip 2001) in various crops, such as cereals, different vegetables and potatoes (e.g. Miles 1942; Parker 1994; Blot and Brunel 1999). Control methods have included repeated tillage, crop rotation, pesticide application and biological control with varying degrees of success (reviewed in Ritter and Richter 2013; Traugott *et al.*^2015^). The progressive banning of chemical insecticides has resulted in an increased focus on biological alternatives for pest control such as the application of entomopathogenic fungi or nematodes (Ritter and Richter 2013). Studies with the entomopathogenic fungus *Metarhizium brunneum* ART2825 Petch (Hypocreales: Clavicipitaceae) have shown promising results in laboratory experiments in controlling *Agriotesobscurus* L.*, A. lineatus* L. and *A. sputator* L. (Kölliker, Biasio and Jossi 2011; Eckard *et al.*^2014^). Improvements in formulation technologies and application strategies or co-applications with botanicals, chemicals or other BCAs have been shown to increase the efficacy of entomopathogenic fungi to control pest insects (Ansari, Shah and Butt 2010; Paula *et al.*^2011^; Behle, Jackson and Flor-Weiler 2013; Kabaluk, Lafontaine and Borden 2015). Formulations have been developed for entomopathogenic fungi in order to protect their spores during storage and distribution of the products, to enhance persistence in the field and/or to facilitate the application process (Glare and Moran-Diez 2016). *Metarhizium* (Metschn.) Sorokin has been formulated based on grains, e.g. sterile barley kernels (Aregger 1992), or was produced in form of microsclerotia (Jaronski and Jackson 2008). Application strategies including pheromone traps or CO_2_ lures have been used to enhance the efficacy of *Metarhizium* spp. against *Agriotes* spp. (Kabaluk, Lafontaine and Borden 2015; Brandl *et al.*^2017^). Also, several natural substances have been tested for controlling *Agriotes* spp. (Ritter and Richter 2013). Among those, garlic was shown to repel and reduce movement of *A. obscurus* larvae, which potentially may enhance the efficacy of *M. brunneum* by weakening the larvae and making them more susceptible to a fungal infection (Eckard *et al.*^2017^).

In this study, we investigated whether applications of the fungus *M. brunneum* ART2825 for controlling *A. obscurus* in potato production affect soil fungal and prokaryotic communities. The study relies on both an experiment in the greenhouse (pots) and a field experiment using different formulations of the fungus and garlic extract as potential efficacy-enhancing agent. Isolation and cultivation on selective medium, simple sequence repeat (SSR) genotyping, and high-throughput amplicon sequencing of ribosomal markers were used to monitor the applied fungus and observe changes in fungal and prokaryotic community structures over a period of 4 months.

## MATERIALS AND METHODS

### Rearing of *Agriotes obscurus* larvae

Lab-reared *A. obscurus* larvae were used for artificial infestation of substrates in the pot experiment. They were reared in a laboratory livestock established by the method of Kölliker, Jossi and Kuske (2009). Briefly, *A. obscurus* adults were collected from the field and placed into pots (ø 30 cm) containing 10–15 L soil rich in humus and were covered with a mesh bag until oviposition. Grass was repeatedly sown into the soil of the pots to guarantee food for the hatched larvae and the pots were kept moist. Five months after establishment, larvae were transferred into a pot containing fresh peat soil with sliced carrots as food source and stored at 10°C in the dark. Four weeks prior to experiments, each larva was placed individually into a cup with moist peat substrate and carrot slices and maintained at 22°C. Only healthy larvae were selected for subsequent infestation of pots.

### Treatments

Nine and five different treatments were applied in six replicates in the pot and the field experiment, respectively. Different treatments and applied doses are listed in Table 1. The entomopathogenic fungus *Metarhizium brunneum* strain ART2825 was either applied as unformulated fungal spore powder (F_powd_) or was formulated as fungus colonized barley kernels (FCBK), as fungal capsules (F_cap_) and as fungal granules (F_gran_). The FCBK were produced in the laboratory as described by Aregger (1992). Batches (1.3 kg) of peeled barley kernels were autoclaved twice in plastic culture bags. Subsequently, the barley kernels were inoculated with culture broth of *M. brunneum* ART2825 in cornsteep medium (diluted to 10^7^ spores/ml with water), which had been incubated at 22°C–24°C for 5 days. Following inoculation, the barley kernels were incubated for 4 to 5 weeks at 22°C–24°C. The F_powd_ was produced by FYTOVITA spol s r. o. (Ostrožská Lhota, Czech Republic) using solid-state fermentation, and it was also used for the F_cap_ which were formulated by M. Przyklenk (University of Applied Sciences, Bielefeld, Germany) according to a modified protocol by Humbert *et al.* (2017). The F_cap_ included 8 × 10^7^ spores/g capsules, autoclaved baker's yeast and calcium alginate. They were formed by dripping *M. brunneum* spore-alginate solution into a crosslinking solution which induced polymerization and formation of beads. The F_gran_, a prototype produced by e-nema GmbH (Schwentinental, Germany), included the same components as the F_cap_; however, an extruder and a fluid-bed dryer were used to form granules. Garlic capsules were produced by S. Gerike (University of Applied Sciences, Bielefeld, Germany) and consisted of 6% garlic oil (Neem Biotech Ltd., Abertillery, UK), calcium alginate, acetic acid and a chitosan coating. Garlic capsules were applied alone but also in combination with FCBK and F_cap_ in order to study potential synergistic effects of *Metarhizium* and garlic. The insecticide clothianidin (Insec; Cheyenne®, Philagro, Saint-Didier-au-Mont-d’Or, France) and sterile barley kernels (BK), which represent the carrier material in the FCBK formulation, were used as positive and negative controls, respectively. The pot experiment included the following nine treatments: FCBK, F_cap_, F_powd_, G_cap_, the combinations FCBK + G_cap_ and F_cap _+ G_cap_, Insec, BK and untreated pots. In the field experiment, five treatments were applied: FCBK, F_cap_, F_gran_, Insec and untreated plots (Table 1).

**Table 1.** Applied doses of the nine and five treatments applied in the pot and field experiment, respectively (n = 6).

Treatment	Amount applied (g/pot or field plot)	
	
	Pot	Field
Fungus colonized barley kernels (FCBK)	5.6	270
Fungal capsules (F_cap_)	7	240
Fungal granules (F_gran_)	NI	240
Fungal spore powder (F_powd_)	0.11	NI
Garlic capsules (G_cap_)	14.4	NI
G_cap_ and FCBK	5.6 + 14.4	NI
G_cap_ and F_cap_	7 + 14.4	NI
Barley kernels (BK)	5.6	NI
Clothianidin (Insec)	0.06	14
Untreated	x	x

The amount of fungal spores in the pot and in the field experiment were 1 × 10^14^ and 5 × 10^13^ spores/ha and clothianidin was applied at a rate of 11 kg/ha. All pots and plots included potato plants and the pest insect.

NI , treatment not included.

### Set-up of the pot experiment

The pot experiment was conducted in a greenhouse at 20°C–25°C from April until September 2014. Each of the nine treatments (Table 1) was replicated six times resulting in 54 pots which were randomly arranged and kept at the same position during the experiment. Pots had a dimension of 22.5 × 25 × 26 cm and two mesh sealed holes (ø 2.5 cm) at the bottom for water drainage and for preventing the escape of *A. obscurus* larvae. Soil (3% humus, 22% clay, 38% silt) with a pH of 7.9 was collected from a field at Agroscope research station Reckenholz, Zürich (Switzerland). The field soil was homogenized with a cement mixer and filled into pots 4 weeks prior to application of the treatments. The pots were kept moist (16 ± 3% water content, no significant difference among treatments), and weeds were removed by hand prior to application of the treatments. Treatments were applied manually onto the soil surface, and then mixed into the upper 15 cm of the soil using a small gardening rake. Subsequently, two pre-sprouted seed potato tubers (*Solanum tuberosum* L.) of the cultivar ‘Celtiane’ were placed in each pot at a depth of 10 cm followed by the release of 10 late instar *A. obscurus* larvae into each pot.

Bulk soil samples were collected from pots before application of treatments and potato tubers on 14 May 2014 (week 0) and post application on 1 July 2014 (week 7) and 26 August 2014 (week 15). Each soil sample consisted of four soil cores (15 cm depth and 1.5 cm width) that were collected crosswise per pot and then mixed. The aboveground potato tissue was cut 5 cm above the soil surface after the third sampling at week 15, at the time when plants became senescent. Two weeks later, the pots were disassembled. Potatoes were harvested and washed, and the damage caused by *A. obscurus*, i.e. the number of holes per tuber, assessed and categorized according to standards provided by the European and Mediterranean Plant Protection Organization (Anonymous 2005). Released *A. obscurus* larvae were re-captured, counted and incubated individually in cups filled with peat substrate and carrot slices as food source at 21°C for 8 weeks to check for infection with *Metarhizium* spp.

### Set-up of the field experiment

The field experiment was performed in an agricultural field located in Mellingen, Switzerland (47°24’24^΄^ N 8°16’12^΄^ E). The soil contained 2% humus, 21% clay and 32% silt at soil pH 7.3. The site is naturally infected with different wireworm species, predominantly of the genus *Agriotes*, and was planted with grass during three seasons preceding the experiment. All cultivation and farming steps were performed by the farmer owning the field except soil sampling, potato planting and potato harvesting. The experimental area was rectangular including 10 blocks with three plots per block (Fig. S1, Supporting Information). Each plot was approximately 3 m wide (four rows of potato plants) and 8.3 m long. The three plots forming a block were connected at the long side and blocks were separated by a 70 cm path. The entire experimental field, including a 3-m wide untreated belt surrounding the plots, measured ∼1600 m^2^.

Bulk soil samples were collected before application of treatments and potato tubers on 21 April 2015 (week 0) and post application on 24 June 2015 (week 9) and 11 August 2015 (week 16). Soil samples were obtained by collecting and combining 10 soil cores (15 cm depth and 2.5 cm diameter) from the inner two rows (five cores from each row) of each plot. One-meter buffer zones at both ends of each plot were not sampled to prevent potential carryover from neighboring plots. The field was ploughed in March 2015 and harrowed once after the first soil sampling in April 2015. Then, treatments were applied manually and integrated into the soil by harrowing for a second time. Subsequently, potato tubers of the cultivar ‘Celtiane’ were planted in rows which were piled immediately after application in order to prevent UV exposure of the products. Fifty-five kilogram per hectare of the fertilizer MgS Ammonsalpeter 25 (Agroline, Roggwil, Switzerland, 25% nitrogen, 5% magnesium, 8.5% sulfur) was applied, and the herbicide Titus (DuPont de Nemours International Sàrl, Le Grand-Saconnex, Switzerland, 25% rimsulfuron) + Exell (Stähler Suisse, Zofingen, Switzerland, 77% detergents, 22% ethylenglycolmonobuthylether), the pesticide Audienz (Omya AG, Oftringen, Switzerland, 44.2% spinosad) and the fungicide Mapro (ISK Biosciences GmbH, Bern, Switzerland, 38,8% fluazinam) were sprayed in May and June. The leaves of potato plants were herbicide treated, after an infection with the fungus *Colletotrichum* Corda had been detected in July, by applying Reglone (Syngenta AG, Basel, Switzerland, 17% diquat) for haulm destruction. After the third soil sampling in August, potato tubers of the inner two rows of each plot were harvested. Fifty potato tubers per plot were randomly selected, washed and *Agriotes*-caused damage scored.

Weather data were obtained from the closest meteorological station in Kuenten CH (6 km from the field site). During the sampling period, the daily mean temperature was 18.3°C and ranged between 7.6°C and 28°C. During this time, a total of 431.8 mm precipitation was recorded. Average humidity was 73.9% and ranged between 53.4% and 97.7%.

### Processing of soil samples, isolation of *Metarhizium* CFU and identification of applied strain

Soil samples were homogenized and sieved with a 5-mm mesh, and aliquots were used for assessment of soil moisture content, for determination and isolation of *Metarhizium* spp. colony forming units (CFU) and for extraction of soil DNA (described below). The CFU determination of *Metarhizium* spp. was performed with slight modifications according to the protocol described by Schneider *et al.* (2012). Three times 20 g of soil per sample were dissolved in 100 ml pyrophosphate solution and plated onto selective medium agar plates resulting in three plates per sample. *Metarhizium* colonies were counted after 10 to 14 days.

After CFU assessment, isolates were selected from the plates for genetic identification using SSR marker-based genotyping. From the pot experiment, five to six isolates were selected from each treatment at week 0, one to two isolates were obtained from all fungal treatments at week 7 and six isolates were chosen from all fungal treatments at week 15. In addition, one to eight isolates per treatment were recovered from *Metarhizium* spp. infected *A. obscurus* larvae which were re-captured after the end of the pot experiment and incubated in the lab for detection of late fungal infections. One *Metarhizium* spp. colony per soil sample ( = plot) per sampling time point was selected from the field experiment.

Fungal tissues isolated from FCBK, F_cap_ and F_gran_ were used as positive controls. Isolates were transferred to potato agar plates and stored at 4°C until all isolates of the pot or the field experiment were collected. Subsequently, all isolates were plated onto filter paper, which was placed on potato agar plates. Mycelium of each isolate was scraped off the filter paper, and DNA was extracted according to the protocol described by Kepler *et al.* (2014). SSR analysis for genotyping of *Metarhizium* isolates was performed using SSR markers Ma2049, Ma2054 and Ma2063 (Set I) and Ma195, Ma307 and Ma2287 (Set V) (Mayerhofer *et al.*^2015^). DNA extracts were diluted 10 to 100 times, and PCR was performed as described in Mayerhofer *et al.* (2015). PCR products were visualized with an ABI 3130xl (Applied Biosystems, Foster City, CA, USA) using 36 cm capillaries and POP-7 polymer. GENESCAN 400 HD ROX was used as an internal size standard. Allele sizes were determined using the software GeneMarker® (SoftGenetics®, State College, PA, USA) and corrected relative to allele sizes of the reference strains *M. anisopliae* ART2062 (Metschn.) Sorokin, *M. brunneum* ARSEF7524 and *M. robertsii* ARSEF7532 J.F. Bisch.

### DNA extraction from soil, PCR and Illumina sequencing

Soil genomic DNA was extracted from each replicate (pot or plot) per treatment and per sampling time point for both experiments. One half gram of each sample was placed into a 2-ml Eppendorf tube containing 0.5 g of glass beads (ø 0.1–0.11 mm; Sartorius, Tagelswangen, Switzerland), vortexed with 1.3 ml extraction buffer and stored at –20°C until further use. Soil DNA was extracted as described by Bürgmann *et al.* (2001) and modified by Hartmann *et al.* (2005). Soil DNA extracts were purified with the NucleoSpin^®^ gDNA clean-up kit (Machery-Nagel, Düren, Germany) and stored at –20°C. DNA concentrations were measured using PicoGreen (Invitrogen, Carlsbad, CA, USA) with a Cary Eclipse fluorescence spectrophotometer (Varian, Inc., Palo Alto, CA, USA) and DNA extracts were diluted to 2 ng/μl with autoclaved dd H_2_0. PCR was adopted from Frey *et al.* (2016) with small modifications. Fungal internal transcribed spacer region 2 (ITS2) was amplified using the primer pair ITS3 (5^΄^ CAHCGATGAAGAACGYRG 3^΄^)/ITS4 (5^΄^ TCCTSCGCTTATTGATATGC 3^΄^) (Tedersoo *et al.*^2014^). The prokaryotic variable region (V3-V4) of the small subunit of the ribosomal RNA (16S rRNA), targeting bacterial and archaeal sequences, was amplified with the modified version of primer pair 341F (5^΄^ CCTAYGGGDBGCWSCAG 3^΄^)/806R (5^΄^ GGACTACNVGGGTHTCTAAT 3^΄^) (Frey *et al.*^2016^). Forward and reverse primers for amplification of ITS2 and V3-V4 included adapter sequences CS1 (forward) and CS2 (reverse) at the 5^΄^ end of each primer to allow multiplexing with the Fluidigm Access Array System (Fluidigm, South San Francisco, CA, USA). Prior to PCR, 20 ng of soil genomic DNA was incubated with 45 μg BSA in 15 μl for 5 min at 90°C. PCR was performed in a volume of 50 μl containing the pre-incubated DNA, 1x PCR buffer containing 15 mM MgCl_2_ (Qiagen, Venlo, Netherlands), 0.4 μM of the forward and the reverse primer, 0.2 mM of each dNTP (Promega, Madison, WI, USA), 1 mM MgCl_2_ (Qiagen), additional 1.8 mg/ml BSA and 2 U of HotStartTaq^®^ Plus DNA polymerase (Qiagen). PCR cycling conditions included one initial denaturation step at 95°C for 5 min, followed by 30 or 35 cycles (for prokaryota or fungi) of denaturation at 94°C for 40 s, annealing at 58°C for 40 s (both primer pairs) and elongation at 72°C for 1 min. PCR was finalized with elongation at 72°C for 10 min. The integrity and quality of the PCR products were checked on an agarose gel. PCR was repeated four times per sample, replicates were pooled and sent for sequencing on a Illumina MiSeq platform at the Génome Québec Innovation Center at the McGill University (Montréal, Canada). There, barcodes were added to the PCR products using Fluidigm Access Array technology to allow multiplex sequencing. Subsequently, PCR products were purified with AMPure XP beads (Beckman Coulter, Brea, CA, USA), and pair-end sequencing was performed using Illumina MiSeq v3 (Illumina Inc., San Diego, CA, USA). Raw sequences were deposited in the NCBI SRA database with the accession number PRJNA386024.

### ITS2 sequence of the applied strain

The sequence of the ITS2 region of *M.brunneum* ART2825 was determined with Sanger sequencing using the primer pair ITS3/ITS4 lacking adapter sequences CS1 and CS2 and the BigDye® Terminator v3.1 cycle sequencing kit (Applied Biosystems). Sequences were visualized using a capillary electrophoresis device (ABI 3130xl Genetic Analyzer, Applied Biosystems) and assembled using DNA Baser 3.4.5 (Heracle BioSoft, Pitesti, Romania).

### Sequence processing and taxonomic classification

Sequences were processed and classified using a customized pipeline (Frey *et al.*^2016^) mostly based on UPARSE within USEARCH v8 (Edgar 2010, 2013). Overlapping paired-end reads were merged using fastq_mergepairs (Edgar and Flyvbjerg 2015) with a minimal overlap of 50 bp and a minimal merge length of 150 bp for fungal and 300 bp for prokaryotic sequences. Substitution errors were removed using the BayesHammer algorithm implemented in SPAdes 3.5 (Nikolenko, Korobeynikov and Alekseyev 2013; Nurk *et al.*^2013^) and primers were removed with Cutadapt 1.8.1 allowing one mismatch (Martin 2011). Quality control was performed using fastq_filter in USEARCH discarding reads with expected total error greater than one (Edgar and Flyvbjerg 2015). Dereplication and clustering into OTUs with 97% identity was performed using derep_fulllength and cluster_otus within USEARCH with concurrent removal of singletons and chimera (Edgar 2013). The eukaryotic or prokaryotic centroids were searched for ribosomal signatures with ITSx (Bengtsson-Palme *et al.*^2013^) or Metaxa2 (Bengtsson-Palme *et al.*^2015^), respectively, and only sequences which included these signatures were kept in the dataset. The algorithm usearch_global was used to map sequences to the centroids (maxdiffs 0, maxaccepts 0, top_hit_only). Eukaryotic sequences were compared to a custom-made NCBI Genbank database (Benson *et al.*^2015^) and the UNITE database (Abarenkov *et al.*^2010^) for taxonomic classification using the naïve Bayesian classifier implemented in MOTHUR v.1.35.1 (Schloss *et al.*^2009^). Sequences that were assigned as *Metazoa*, *Viridiplantae*, *Protista* and unclassified were removed from the dataset. The GREENGENES database (DeSantis *et al.*^2006^; McDonald *et al.*^2012^) was used for taxonomic classification of prokaryota. Subsequently, only archaeal and bacterial sequences were kept in the dataset.

### Statistical analyses

The abundance of *Metarhizium* spp. was assessed by counting CFU and calculating CFU g^−1^ soil dry weight. *Metarhizium* CFU g^−1^ soil dry weight in three replicates per soil sample were averaged using the median per sample. Significance of differences was assessed with a Kruskal–Wallis rank sum test (Hollander and Wolfe 1973) followed by Dunn's Kruskal-Wallis multiple comparison test in the FSA package (Dunn 1964; Ogle 2016) with Benjamini-Hochberg (BH) *P*-value adjustment (Benjamini and Hochberg 1995) implemented in R version 3.3.0 used with Rstudio version 0.98.994 (R-Development-Core-Team 2008; RStudio-Team 2015). Correlations were calculated using the Pearson correlation coefficient in R. Efficacy of the treatments in the pot experiment was determined by calculating the % control based on percentage of undamaged potato tubers compared to the control (Abbott 1987). Saturation of sequencing was checked using intrasample rarefaction curve analysis (rarefaction.single in MOTHUR) with a re-sampling without replacement approach and plotted in R. Observed OTU richness and the inverse Simpson index representing effective number of species of soil fungal and prokaryotic communities were calculated with ‘summary.single’ in MOTHUR (Simpson 1949; Jost 2006). This includes an iterative subsampling procedure (9999 times) to the sampling depth of the sample with the fewest sequences (pot experiment: 7425 fungal and 9088 prokaryotic sequences, field experiment: 2101 fungal and 10 896 prokaryotic sequences). Dissimilarities in the fungal or prokaryotic communities between pairs of samples were assessed using Bray-Curtis (BC) dissimilarity matrices with iterative subsampling (9999) which were calculated with dist.shared in MOTHUR. Significance of differences of the fungal and prokaryotic communities among treatments and sampling time points was assessed with overall and pairwise ANOSIM (Spearman rank correlation and 9999 iterations) based on BC dissimilarities implemented in PRIMER v7 (Clarke 1993; Clarke and Gorley 2015) and with overall PERMANOVA based on BC dissimilarities using the function adonis within the R package vegan (Oksanen *et al.*^2016^) followed by assessment of pairwise differences using the function pairwise.perm.manova within the R package RVAideMemoire (Hervé 2017). Unconstrained ordinations were determined in R using non-metric multidimensional scaling (NMDS) based on BC dissimilarities with the function metaMDS within the R package vegan (Faith, Minchin and Belbin 1987; Minchin 1987; Oksanen *et al.*^2016^). Significant differences of relative sequence abundance of each OTU across sampling time points, treatments and interactions among sampling time points and treatments were assessed with PERMANOVA based on Euclidian distance using the function adonis followed by BH *P*-value correction. Pairwise differences were calculated for each OTU with a significant overall PERMANOVA pseudo F-statistic per sampling time point using the R function pairwise.perm.manova. In addition, the contribution of single OTUs to BC dissimilarities was calculated using the SIMPER (similarity percentage) routine in Primer 7v (Clarke 1993) with the 100 most abundant OTUs (relative abundance and square root transformation) per sampling time point. Only significant pairwise comparisons of a treatment and the control assessed with pairwise ANOSIM and pairwise PERMANOVA were selected for the SIMPER analyses.

## RESULTS

### Abundance of the applied *Metarhizium* strain and efficacy of biocontrol treatments in pots

**Figure 1.**
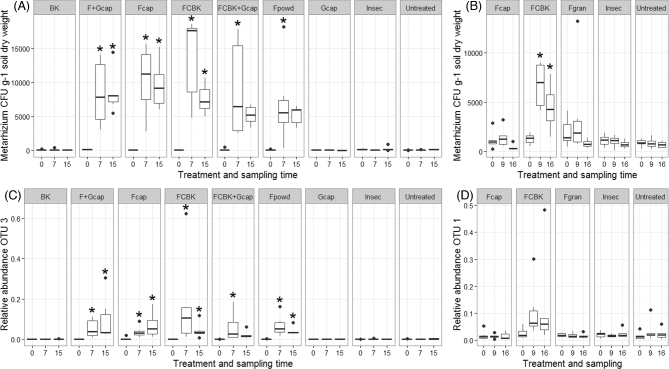
Abundance of *Metarhizium* spp. CFU g^−1^ soil dry weight per treatment and sampling time in the pot (**A**) and the field (**B**) experiment and abundance of the OTU including the sequence of the applied strain per treatment and sampling time in the pot (**C**) and field experiment (**D**). Asterisk indicates significant differences to untreated control at the corresponding sampling time point (n = 6; *P* ≤ 0.05).

The abundance of *Metarhizium* spp. increased significantly in all fungus-treated pots from a median of 56–144 CFU g^−1^ soil dry weight before application to 5569–17 596 CFU g^−1^ soil dry weight in BCA-treated pots at week 7 and remained high until the end of the experiment (Fig. 1A). In contrast, the abundance of *Metarhizium* spp. in pots not treated with the fungus remained low with a median of 0–153 CFU during the entire experiment.

**Figure 2.**
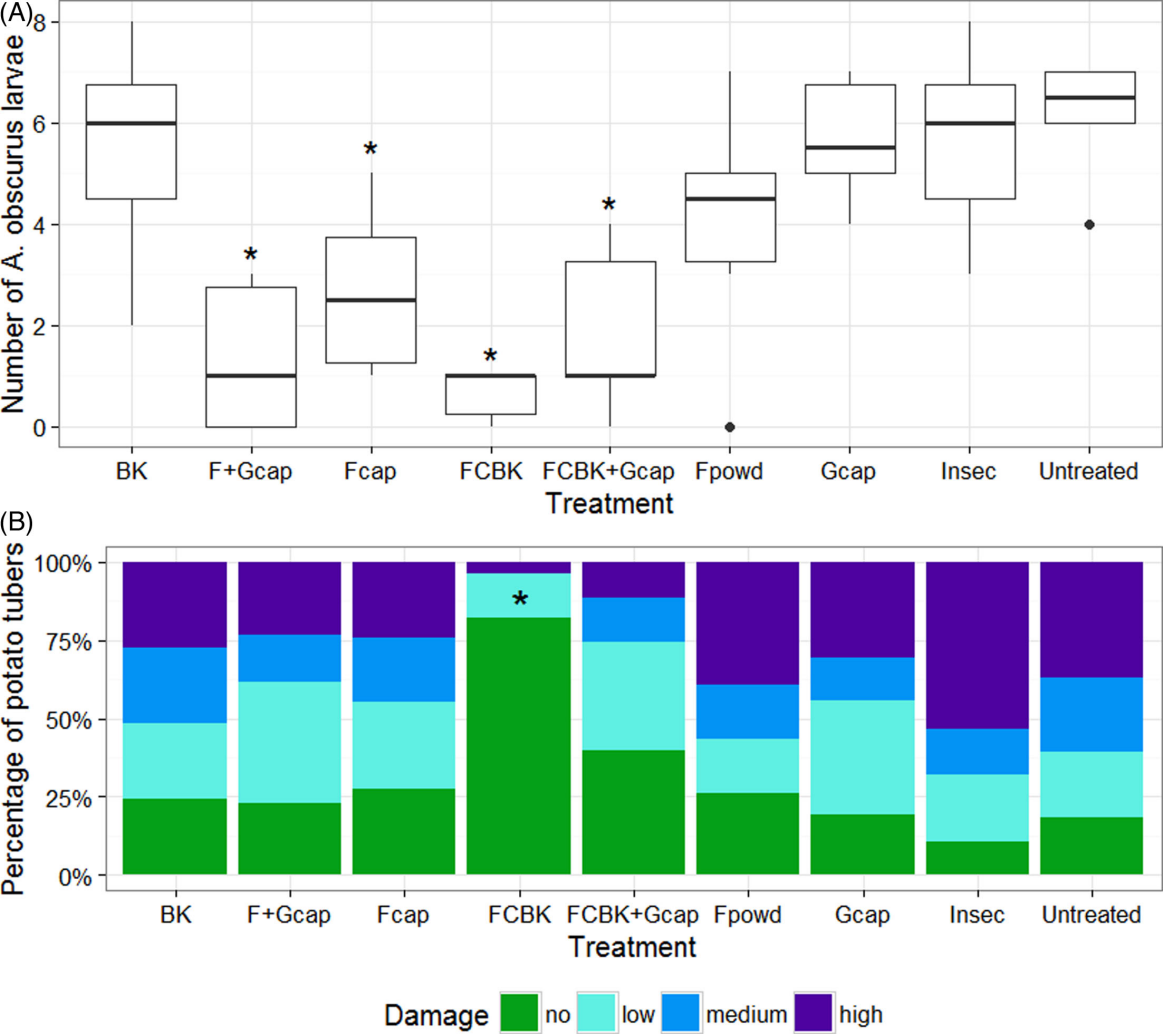
Number of *A. obscurus* larvae per treatment retrieved from pots initially receiving 10 larvae (**A**) and levels of potato tubers damage in percentage of total number of tubers harvested per treatment (**B**): no (0 holes per tuber; SE = 7%–13%), low (1–2 holes per tuber; SE = 4%–10%), medium (3–5 holes per tuber; SE = 0%–10%), high (>4 holes per tuber; SE = 4%–14%), n = 2 to 10 potato tubers per pot. Asterisk indicates significant differences to untreated control (*P* ≤ 0.05).

SSR marker-based genotyping revealed that 92% of the isolates (n = 39) selected from soil of fungus-treated pots after applications had the genotype of the applied strain (Table S1, Supporting Information). The applied strain was already detected in pot substrates before treatment (week 0), but only one out of five isolates from control pots and two out of six isolates from pots treated with F_cap _+ G_cap_ revealed the genotype of the applied strain. The abundance of *Metarhizium* spp. in these pots was 57 and 141 CFU g^−1^ soil dry weight. Except for F_powd_-treated pots, significantly fewer *Agriotesobscurus* larvae were retrieved from fungus-treated pots, as compared to the untreated controls at the end of the experiment (Fig. 2A). A median re-capture rate of six *A. obscurus* larvae out of ten released ones in the untreated pots was within the range of what was observed in previous experiments (unpublished data). The lowest number of *A. obscurus* larvae was found in the FCBK-treated pots with a median of one larva per pot. From 18 mycosed *A. obscurus* cadavers obtained from pots treated with FCBK, F_cap_, F_powd_, FCBK + G_cap_ and F_cap _+ G_cap_ 82.4% were infected by the applied strain (Table S1; for information on treatments, see Table 1). One *A. obscurus* larva originating from an Insec-treated pot was also infected with the applied strain. Treatments with G_cap_, BK or Insec did not result in decreased numbers of *A. obscurus* larvae and the combinations of FCBK or F_cap_ with G_cap_ did not enhance efficacy of treatments (no further decrease in the number of *A. obscurus* larvae). The number of *A. obscurus* larvae was moderately and significantly correlated with the percentage of damaged potatoes (r = 0.46, *P* < 0.001). The mean percentage of undamaged potato tubers ranged from 10% to 81% (Fig. 2B). FCBK was the only treatment resulting in a significantly higher number of undamaged potato tubers as compared to the control (Fig. 2B) yielding an efficacy (undamaged potatoes compared to the control) of 77%. There were no significant differences in percentages of low, medium or highly damaged potato tubers among treatments. The combined treatments of fungus and garlic, G_cap_, BK and Insec did not exhibit an effect on potato tuber damage.

### Abundance of the applied *Metarhizium* strain and efficacy of biocontrol treatments in the field

Abundance of *Metarhizium* spp. increased after the application of FCBK from a median of 1304 to 6969 CFU g^−1^ soil dry weight 9 weeks after application and slightly decreased to 4261 CFU g^−1^ soil dry weight at week 16 (Fig. 1B). The application of F_gran_ or F_cap_ did not yield significantly increased *Metarhizium* abundances. The applied strain was not detected in any of the treatments at week 0 as shown by SSR-based genotyping (Table S1). In all fungus-treated field plots, 36% of the isolates (n = 36) had the genotype of the applied strain; however, after the application of FCBK 91.7% of the isolates (n = 12) were identified as the applied strain, whereas after the application of F_cap_ and F_gran_ only 0 and 16.7% of the isolates (n = 12) had the genotype of the applied strain. Of the 50 potato tubers analyzed per plot, a median of 42% to 51% was damaged and the number of damaged potato tubers did not differ among treatments (Fig. S2, Supporting Information).

### Soil microbial communities of the pots

After quality filtering, a mean of 22 406 ± 8505 fungal and 19 706 ± 3418 prokaryotic sequences per sample was obtained in the pot experiment and clustered into a mean of 433 ± 51 fungal and 2795 ± 245 prokaryotic OTUs per sample, respectively. Rarefaction curve analysis revealed that fungi were sampled more exhaustively but with a higher variation among samples than prokaryota (Fig. S3A and B, Supporting Information). The fungal community across all pots was dominated by Ascomycota (84%) followed by Zygomycota (7%), Basidiomycota (6%), Chytridiomycota (1%), Glomeromycota (0.4%) and Blastocladiomycota (0.004%), besides unclassified fungi (0.01%; Fig. S3C). A total of 45 bacterial and three archaeal phyla were detected across all pots. The most abundant phyla (>10%) were Proteobacteria (22%), Actinobacteria (20%), Chloroflexi (14%) and Acidobacteria (13%; Fig. S3D).

### Abundance of the applied strain and effects of treatments on soil microorganisms in pots

Within the fungal sequence dataset of the pot experiment, two OTUs were assigned to the genus *Metarhizium*. OTU 3 and OTU 1703 were classified as *M.brunneum* and *M.flavoviride* var*. flavoviride* W. Gams & Rozsypal with a sequence abundance of 87 207 and 14, respectively. OTU 3 occurred in 158 of the 162 samples and included 2641 unique sequences. The most abundant unique sequence (33 693 sequences) exactly matched the ITS2 sequence of the applied strain (GenBank Acc. N. KY786031). The abundance of OTU 3 was significantly increased in all *M.brunneum*-treated pots at week 7 and 15, and this increase correlated with the increase of *Metarhizium* spp. CFU g^−1^ soil dry weight (r = 0.65, n = 162, *P* < 0.001; Fig. 1A and C). OTU 3 was removed from the fungal dataset in order to avoid analytical bias of the abundance of OTU 3 on statistical tests used to assess treatment effects on the community structure of soil fungi.

**Figure 3.**
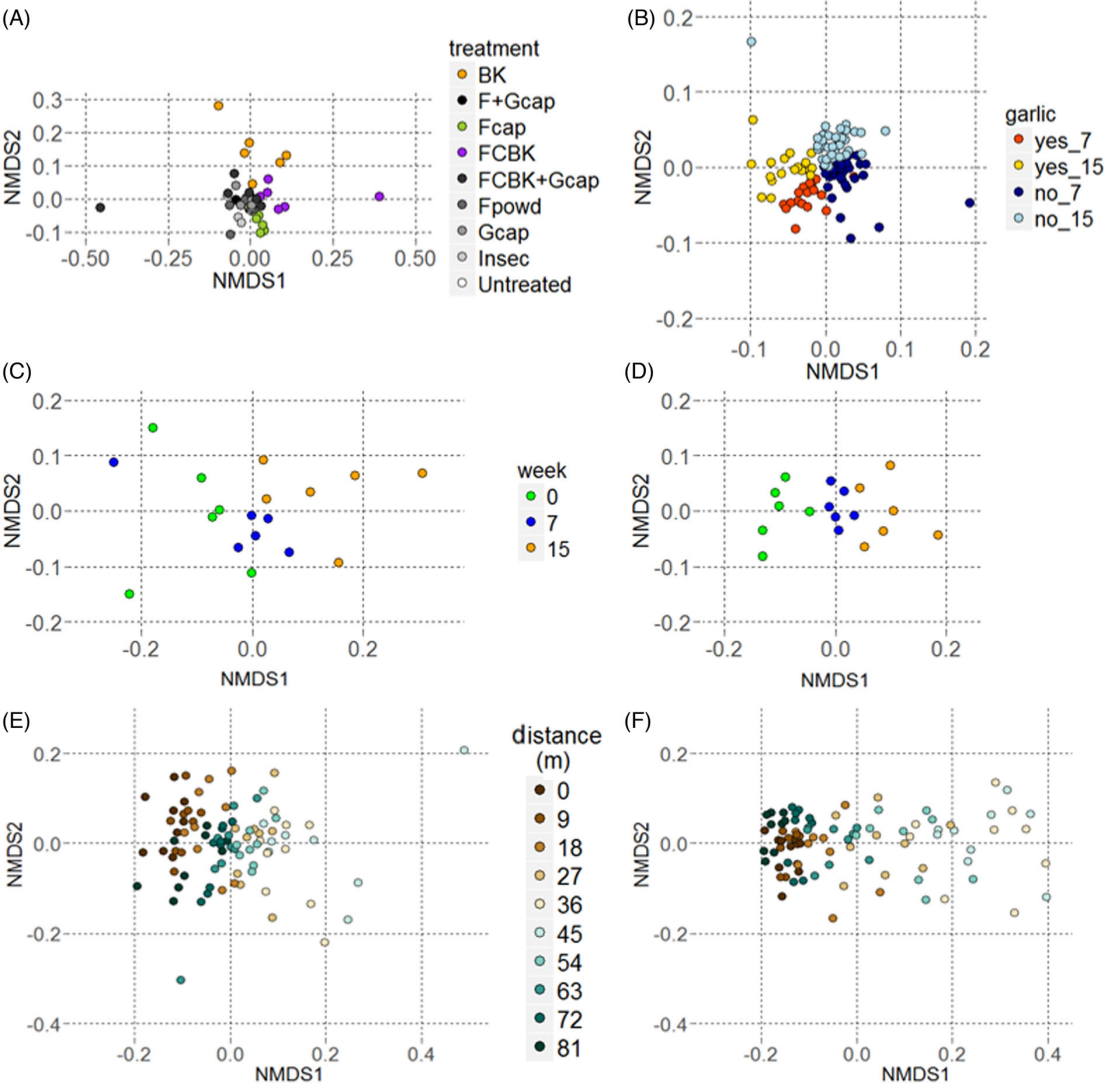
Unconstrained ordination of soil samples based on Bray-Curtis dissimilarities of fungal communities per treatment at week 7 in the pot experiment (**A**, stress = 0.14), of prokaryotic communities for treatments with and without garlic at week 7 and 15 in the pot experiment (**B**, stress = 0.2), of fungal (**C**, stress = 0.11) and prokaryotic (**D**, stress = 0.13) communities in the untreated pots at different sampling time points and of fungal (**E**, stress = 0.2) and prokaryotic (**F**, stress = 0.09) communities along a spatial gradient across the long side of the field.

OTU richness of the fungal communities did not differ among treatments at week 0 and at week 15. However, at week 7, OTU richness was significantly lower in BK and the FCBK-treated pots as compared to the untreated pots at week 7 (Fig. S4A, Supporting Information). No significant differences in OTU richness were observed among prokaryotic communities of the different treatments compared to untreated pots at the respective sampling time points (Fig. S4B). Similar results were obtained using the inverse Simpson index (data not shown). Overall ANOSIM analyses based on BC dissimilarities of the fungal communities across all pots revealed no differences among treatments at week 0; however, small but significant differences were detected at weeks 7 and 15 (Table 2). Pairwise ANOSIM tests of treatments compared to untreated pots and the NMDS analyses revealed that the fungal communities were moderately affected (R > 0.4) by the addition of BK, FCBK and F_cap_ at week 7 (Table S2, Supporting Information; Fig. 3A). Also, the fungal communities in these three treatment groups differed among each other (mean pairwise ANOSIM R-value of 0.62 ± 0.09) at week 7 (Table S2). At week 15, the fungal communities in pots treated with BK, FCBK, FCBK + G_cap_, F_cap_ and F_cap _+ G_cap_ differed significantly from the untreated pots and among each other (mean pairwise ANOSIM R-value of 0.41 ± 0.09; Table S2). While prokaryotic communities in the pots did not differ among the treatments at week 0, small changes were detected at week 7 and week 15 (Table 2). Pairwise comparisons of the prokaryotic communities of different treatments compared to untreated pots at the respective sampling time point and the NMDS plot revealed that all treatments including garlic (G_cap_, FCBK + G_cap_, F_cap _+ G_cap_) affected the prokaryotic communities at week 7 and 15 (Table S2, Fig. 3B). However, there were no differences in pairwise ANOSIM

 

comparisons among the three garlic treatments (Table S2). Corresponding results were obtained for overall and pairwise analyses of fungal and prokaryotic communities using PERMANOVA (Table S3, Supporting Information).

**Table 2.** Differences in the fungal and the prokaryotic community structures among treatments at different sampling time points (n = 6), treatments with (n = 18) and without garlic (n = 36), of untreated pots or plots (n = 6) over time and among blocks across the long side of the field (n = 9) in the pot and/or field experiment assessed with overall ANOSIM (analysis of similarity) based on Bray Curtis dissimilarity.

		Fungi	Prokaryota
Experiment	Overall test	ANOSIM R	ANOSIM R
Pot	Among treatments at week 0	0.03	0.08^*^^*^
Pot	Among treatments at week 7	0.31^*^^*^^*^	0.38^*^^*^^*^
Pot	Among treatments at week 15	0.26^*^^*^^*^	0.29^*^^*^^*^
Pot	Untreated over time	0.22^*^^*^	0.63^*^^*^^*^
Field	Among treatments at week 0	–0.1	–0.09
Field	Among treatments at week 9	0.04	–0.05
Field	Among treatments at week 16	0.02	–0.08
Field	Untreated over time	0.12^*^	–0.04
Field	Among 10 blocks along the field	0.49^*^^*^^*^	0.61^*^^*^^*^

^*^ *P* ≤ 0.05

^*^^*^ *P* ≤ 0.01

^*^^*^^*^ *P* ≤ 0.001

NA , not assessed.

**Figure 4.**
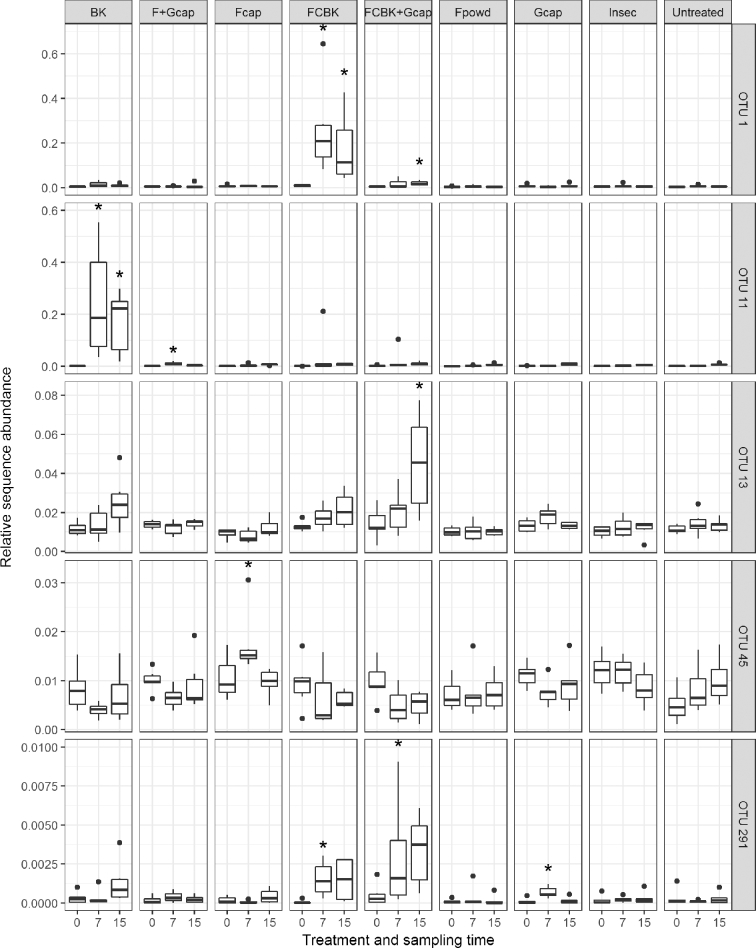
Relative sequence abundance of fungal OTUs among different treatments and time points (n = 6). Asterisk indicates a significant difference between treatments and untreated pots at the respective sampling time point (*P* < 0.05). OTU 1, OTU 11, OTU 13 and OTU 45 were classified as Bionectriaceae, *R.oryzae*, Nectriaceae and *Mortierella* spp., respectively. OTU 291 was an unclassified fungal OTU.

Assessing differences in relative sequence abundance of each fungal OTU among treatments revealed only 0.2% (7) of the fungal OTUs with a significant overall PERMANOVA pseudo F-value for the factor treatment. The relative abundance of five of these seven fungal OTUs changed significantly between untreated and either BK, FCBK, F_cap_, FCBK + G_cap_ or F_cap _+ G_cap_ assessed with pairwise PERMANOVA (Fig. 4). Similarity percentage analyses (SIMPER) based on BC dissimilarities were performed to identify fungal and prokaryotic OTUs contributing to differences of microbial community structures of single treatments and untreated control pots with a significant pairwise comparison assessed with ANOSIM and PERMANOVA (Tables S4 and S5, Supporting Information). Data from PERMANOVA and SIMPER analyses revealed that fungal OTU 1, which was classified as member of the family *Bionectriaceae*, increased significantly in the FCBK-treated pots at week 7 and in the FCBK and FCBK + G_cap_-treated pots at week 15 (Fig. 4) and contributed 12.3% and 10.6% to the differences between FCBK-treated pots and untreated pots at week 7 and 15, and 2% to the differences between FCBK + G_cap_ -treated and untreated pots at week 15 (Table S4). Fungal OTU 11, classified as *Rhizopus oryzae*, increased significantly in the BK-treated pots at week 7 and in the BK and F_cap _+ G_cap_-treated pots at week 15 (Fig. 4) and accounted for 12% and 9.6% of the differences between BK-treated and untreated pots at week 7 and week 15 and 0.8% of the differences between F_cap _+ G_cap_-treated and untreated pots at week 15. Fungal OTU 13, which was identified as member of the family Nectriaceae, increased significantly in FCBK + G_cap_-treated pots at week 15 (Fig. 4) and contributed 2.8% to the differences between FCBK + Gcap -treated and untreated pots at week 15. Fungal OTU 45, identified as *Mortierella* spp., increased significantly in F_cap_-treated pots at week 7 (Fig. 4) and contributed 1.5% to the differences between F_cap_-treated and untreated pots at week 7 (Table S4). The unclassified fungal OTU 291 increased significantly in FCBK, FCBK + G_cap_ and G_cap_-treated pots at week 7 (Fig. 4); however, it was not among the 100 most abundant OTUs which were used for the SIMPER analyses.

Overall, PERMANOVA of relative sequence abundance per OTU revealed that 0.46% (44) of the prokaryotic OTUs were significantly affected by treatments (data not shown) and of these 36 were significantly different between any treatment and untreated pots. None of these 36 OTUs were significantly different between untreated and FCBK or F_powd_-treated pots, two changed significantly in pots treated with F_cap_, one changed significantly after the addition of Insec and 33 were significantly different between untreated and any treatment including garlic (Fig. S5, Supporting Information). Only 10 of the OTUs detected with PERMANOVA were among the 100 most abundant OTUs investigated with SIMPER analyses, and they contributed between 0.4% and 5.41% to the respective differences (Table S5).

### Changes of the microbial communities over time in pots

OTU richness of fungal communities in the untreated pots did not change over time. In contrast, OTU richness of soil prokaryotic communities in the untreated pots increased significantly and continuously from week 0 to week 15 (Fig. S4A and B, Supporting Information). Similarly, a significant increase of prokaryotic OTU richness was observed in BK, F_cap _+ G_cap_, FCBK + G_cap_, F_powd_ and Insec-treated pots and it tended to increase also in all other treatments (F_cap_, FCBK and G_cap_). Overall ANOSIM values, overall PERMANOVA and NMDS revealed that fungal and prokaryotic community structures of the untreated pots differed among the sampling time points (Table 2, Fig. 3C and D, Table S3). Fungal community structures in the untreated pots changed slightly but significantly between week 0 and 15 (pairwise ANOSIM R = 0.45, *P* = 0.006), and similar changes over time were observed in FCBK, BK, F_cap_ and F_cap _+ G_cap_-treated pots (mean pairwise ANOSIM R-value of 0.42 ± 0.13; Table S2). The prokaryotic communities of the untreated pots underwent a continuous significant shift across the three sampling time points which was shown by NMDS (Fig. 3D) and pairwise ANOSIM comparisons of week 0 to 7 and 0 to 15 resulting in R-values of 0.65 and 0.43, respectively (Table S2). Corresponding significant changes over time of the prokaryotic community structures were observed in all treated pots (mean pairwise ANOSIM R-values of 0.63 ± 0.16 week 0 to 7 and 0.4 ± 0.14 week 7 to 15; Table S2). Assessing differences in relative sequence abundance of fungal and prokaryotic OTUs showed that 99 fungal and 776 prokaryotic OTUs were significantly affected by time (data not shown).

### Soil microbial communities in the field

A mean of 19 610 ± 12 252 fungal sequences per sample were obtained for 89 field samples (excluding one sample with a sequence abundance of only 360) and clustered into a mean of 435 ± 98 OTUs per sample. The 90 field samples included a mean of 17 322 ± 2437 prokaryotic sequences which were clustered into 1767 ± 132 OTUs per sample. Rarefaction analyses revealed that sampling the fungal diversity was closer to saturation than the prokaryotic sampling; however, variation was lower among prokaryotic samples (Fig. S6A and B, Supporting Information). The following six fungal phyla were detected in descending abundance in the soil of the field experiment: Ascomycota (79%), Basidiomycota (11%), Zygomycota (4%), Chytridiomycota (1%), Glomeromycota (0.7%) and Blastocladiomycota (0.03%) with 0.2% unclassified fungal sequences (Fig. S6C). Forty-five bacterial phyla were detected across the field samples. Bacterial phyla with an abundance of at least 10% comprised Proteobacteria (23%), Actinobacteria (17%), Chloroflexi (11%), Verrucomicrobia (11%) and Planctomycetes (11%) (Fig. S6D). The archaeal phylum Crenarchaeota (3%) was the only one of three archaeal phyla representing more than 1% prokaryotic sequences.

### Abundance of the applied strain and effects of treatments on microbial communities in the field

Three OTUs were classified as *Metarhizium* within the fungal sequence dataset of the field samples. OTU 1 (including 73 521 sequences), OTU 2930 (including 3 sequences) and OTU 2732 (including 4 sequences) were assigned to *M. brunneum, M. anisopliae* and *Metarhizium* spp., respectively. OTU 1 included 4871 unique sequences, and the unique sequence which exactly matched the ITS2 region of the applied strain was detected 6735 times (data not shown). The relative abundance of OTU 1 was significantly higher in FCBK-treated field plots 9 and 16 weeks after the treatment (Fig. 1D). None of the other treatments resulted in increased OTU 1 abundance. There was a positive correlation between the relative abundance of OTU 1 and the number of *Metarhizium* spp. CFU g^−1^ soil dry weight (r = 0.66, n = 90, *P* < 0.001). OTU 1 was deleted from the fungal dataset in order to avoid analytical bias on statistical tests when assessing changes in fungal communities. There were no significant differences in OTU richness or the inverse Simpson index of the fungal and prokaryotic communities among treatments at different sampling time point (data not shown). Overall ANOSIM and pairwise PERMANOVA showed that neither fungal nor prokaryotic communities in the field were affected by the treatments compared to the untreated plots at the respective sampling time points (Table 2, Table S6, Supporting Information).

### Changes of the microbial communities over time and space in the field

Fungal and prokaryotic communities in the untreated plots did not differ in their OTU richness and inverse Simpson index (data not shown), and in their community structures based on BC dissimilarities over time assessed with ANOSIM (Table 2). However, community structure analyses based on BC dissimilarities assessed with overall PERMANOVA revealed a significant time effect on fungal and prokaryotic communities (Table S6). ANOSIM and PERMANOVA analyses revealed significant spatial effects (Table 2, Table S6). Fungal and prokaryotic communities both changed gradually from one end to the middle of the field (about 45 m) and became similar again towards the other end of the field (Fig. 3E and F). Fungal OTU richness differed significantly between the middle (36 and 45 m) and the end of the field (72 and 81 m, Fig. S4C). The community structure (based on BC dissimilarities and visualized by NMDS) of fungal communities differed among blocks (including three plots each) along the long side of the field, i.e. among blocks from the middle section of the field compared to blocks from both ends (Table 2, Fig. 3E, Table S2). Corresponding spatial changes were also detected for the prokaryotic community structures (Table 2, Fig. 3F; Fig. S4D, Table S2).

## DISCUSSION

Risk assessment of any environmental hazard, i.e. an agent or activity causing a hazard, includes the assessment of exposure to the hazard and effects on the population or individual exposed to the hazard (Brown 1985; U.S. Interagency Staff Group on Carcinogenesis 1986). In this study, exposure was defined as a significant increase of *Metarhiziumbrunneum* ART2825 abundance. Exposure analysis was performed with a cultivation-dependent approach (i.e. determination of *Metarhizium* spp. CFU followed by identification of the genotype of the applied strain) and with a cultivation-independent approach (i.e. assessment of the OTU of the applied strain within the amplicon sequences). With both approaches, significant exposure to the applied fungal strain was demonstrated both in the pot experiment and in FCBK-treated field plots. Isolates of the genotype of *M. brunneum* ART2825 were detected at low frequency (6%) in pots before application (untreated and F_cap _+ G_cap_) and isolated from a larvae from an Insec-treated pot and very likely represent natural occurrence of the strain, since the soil used in the pot experiment originated from a field at Agroscope Reckenholz where *M. brunneum* ART2825 has originally been isolated from an *Agriotesobscurus* larva (Kölliker, Biasio and Jossi 2011; Eckard *et al.*^2014^). Although the applied strain established in all fungal-treated pots and in FCBK-treated plots, the biocontrol effect was limited. Only the application of FCBK lead to a 77% efficacy (increase of undamaged potato tubers compared to the control) and a significant reduction of *A. obscurus* larvae in the pot experiment, which corroborated previous laboratory experiments (Kölliker, Biasio and Jossi 2011; Eckard *et al.*^2014^). The number of *A. obscurus* larvae was significantly reduced in F_cap_, F + G_cap_ and FCBK + G_cap_-treated pots compared to untreated pots; however, this did not result in reduced potato tuber damage. The inconsistent results of potato tuber damage and number of *Agriotes* larvae might result from feeding interruptions prior and post molting, which may be uncoordinated within a population (Furlan 1998, 2004; Sufyan, Neuhoff and Furlan 2014) and differences in foraging behavior of *A. obscurus* larvae possibly due to different volatile organic compounds (reviewed in Barsics *et al.*^2014^) emitted from treatments. In contrast to the pot experiment, no biocontrol success was achieved in the field in any of the treatments within one season of fungal applications. This might be explained by unfavorable conditions for the fungus possibly created by non-optimal soil moisture, soil texture, soil temperature or antagonistic microbes (Jaronski 2007). In addition, the applied strain may not be able to provide sufficient protection against all *Agriotes* species present in the field that have been shown to be difficult to control (Blackshaw and Vernon 2008; Sufyan, Neuhoff and Furlan 2013; Sufyan, Neuhoff and Furlan 2014). In other field studies using *Metarhizium* spp. to control *Agriotes* larvae, varying degrees of success have been reported (Kabaluk *et al.*^2005^; Ritter, Katroschan and Richter 2011). For instance, *M. brunneum* ART2825 formulated as FCBK was applied to protect lettuce from *A. sputator* and *A. ustulatus* and showed 21% and 65% reduction of the two pest insects, respectively (Ritter, Katroschan and Richter 2011). However, insignificant reduction in potato tuber damage was detected after the application of *M. anisopliae* granules (Kabaluk *et al.*^2005^). In the pot trial of this study, the combined treatments of *M. brunneum* ART2825 and garlic capsules (FCBK + G_cap_, F_cap _+ G_cap_) did not enhance efficacy, an observation which was also made in a laboratory experiment using two-dimensional terraria (Eckard *et al.*^2017^). One reason for insufficient control of *Agriotes* larvae may be a repelling effect of *Metarhizium* spp. on *Agriotes* spp. (Kabaluk *et al.*^2005^). The use of attractants such as CO_2-_emitting capsules or pheromone pitfalls, as tested in other studies, may help to overcome possible repelling effects (Kabaluk, Lafontaine and Borden 2015; Brandl *et al.*^2017^). The application of the insecticide clothianidin was neither successful in the pot experiment nor in the field experiment. This is in accordance with results obtained from bioassay experiments, where *A. obscurus* larvae have been exposed to clothianidin-treated wheat seedlings (van Herk *et al.*^2008^). In this bioassay, over 70% of the larvae were moribund following a similar insecticide treatment but most recovered 14 days after application. However, even though efficacy of the treatments was limited in our study, criteria for exposure were nevertheless achieved and allowed an assessment of effects of *M. brunneum* ART2825 on soil microorganisms in the pot and in the field experiment.

Application of *M. brunneum* ART2825 formulated as FCBK and F_cap_ (but not the application of fungal spore powder alone) resulted in slight changes of the fungal communities in the pot trial, suggesting that the observed effects on microbial communities were caused by compounds of the formulations rather than by the fungus itself. These small changes between untreated pots and FCBK-treated pots were reflected in a significant increase of only two OTUs which were classified as a member of Nectriaceae and an unclassified fungus. The taxonomic classification of these OTUs allowed very limited assumptions of their functions and possible interactions with the applied strain. The changes between untreated pots and pots treated with F_cap_ were reflected in a significant increase of only one OTU classified as *Mortierella* spp. which are known for their saprophytic life style. This fungus may profit from the alginate carrier; however, it increased with a strong variation as observed in most treatments and sampling time points. In a similar pot experiment, aimed at controlling *Diabrotica v. virgifera* LeConte, the application of FCBK and similar fungal capsules did not affect the fungal communities (J. Mayerhofer in preparation). This may suggest that the impact of FCBK and F_cap_ application on fungal communities is context dependent involving also soil specific or environmental factors. Application of FCBK had also no effect on fungal and prokaryotic communities in the field, and all fungal treatments had no effect on prokaryotic communities in the pot experiment. Our results are in agreement with other studies involving entomopathogenic fungi, e.g. *M.anisopliae* or *Beauveria bassiana* (Bals.-Criv.) Vuill., which detected no, small or only transient effects on soil microorganisms (Hu and St Leger 2002; Rai and Singh 2002; Kirchmair *et al.*^2008^; Schwarzenbach, Enkerli and Widmer 2009; Hirsch *et al.*^2013^). Likewise, the release of other microorganisms for control of phytopathogens, weeds or nematodes resulted in small or transient effects on soil microbial communities (Grosch *et al.*^2006^; Rousidou *et al.*^2013^; Zimmermann *et al.*^2016^). Furthermore, microorganisms released as biofertilizers, phytostimulators or plant growth promotors had no effects on bacterial communities in the rhizosphere (Lerner *et al.*^2006^; García de Salamone *et al.*^2010^; Kröber *et al.*^2014^) or only moderate effects on bacteria and fungi in the rhizosphere or the bulk soil (van Dillewijn, Villadas and Toro 2002; Trabelsi *et al.*^2011^; Schmidt *et al.*^2012^).

The untreated control pots and field plots allowed to assess changes of the microbial communities over time including seasonal and environmental changes, and effects caused by the plants on the resident microbial soil communities. In our study, time-related effects on the fungal and the prokaryotic soil communities in the pot experiment were similar or greater than the treatment effects. This is in agreement with other studies showing that seasonal changes of soil microbial composition in relation to developmental stage of the plant exceed treatment effects of applied fungi and bacteria in bulk soil (Savazzini, Longa and Pertot 2009) as well as in the rhizosphere in the field (van Dillewijn, Villadas and Toro 2002; Grosch *et al.*^2006^; Zimmermann *et al.*^2016^). In the field experiment of our study, the assessment of temporal changes using ANOSIM and overall PERMANOVA showed contradicting results which may have resulted from different sensitivities of the tests. However, the fungal and prokaryotic community structures varied spatially across the field. We suspect that this variation may be related to differences in edaphic factors across the field. Humus, clay and silt content as well as soil pH were assessed, but results did not yield sufficient resolution to support this hypothesis. In other studies, soil edaphic factors including pH, organic carbon, texture, soil moisture and land management have been shown to influence soil microorganism at the agricultural plot scale (Chen *et al.*^2007^; Philippot *et al.*^2009^; Rousk *et al.*^2010^; Naveed *et al.*^2016^).

Entomopathogenic fungi are formulated for applications in order to increase persistence, efficacy or shelf life of the fungi (Burges 1998). *Metarhiziumbrunneum* ART2825 was applied in form of FCBK, F_cap_ and F_powd_ in the pot experiment. The addition of BK, the non-fungal component of the FCBK, also affected fungal communities. These effects were mainly due to an increased abundance of *Rhizopus oryzae,* a well-known degrader of organic matter. The increase of the relative sequence abundance of *R.oryzae* varied among replicates as shown by a large dispersion which may indicate that the response of soil microbial communities was pot specific over time and may indicate the introduction of responsive microorganism by the addition of potato tubers. Surprisingly, *R.oryzae* was not enhanced in the FCBK-treated pots. Possibly, because the niche ‘BK’ was already occupied by the applied strain preventing *R. oryzae* to colonize this nutrient source. The results of this study suggest that the formulations may have been responsible for these effects. Similarly, the effects caused by a biological nematicide containing the fungus *Paecilomyces lilacinus* (Thom) Samson formulated with glucose and skimmed milk were triggered by the formulation only (Rousidou *et al.*^2013^). The prokaryotic communities reacted to the application of G_cap_ and the combinations of FCBK + G_cap_ and F_cap _+ G_cap_ in a very similar way but not after application of fungal products only, suggesting that the observed effects were due to the application of G_cap_. The effects of G_cap_ on soil prokaryotes resulted either from garlic oil, parts of the formulation (not studied separately) or the combination of both. Garlic has been used traditionally as an antimicrobial agent in medicine and for human consumption, but more recently also to protect plants against soil-borne fungal and bacterial diseases (Lawson 1998; Curtis *et al.*^2004^; Sealy, Evans and Rothrock 2007). A possible mechanism explaining the effect of garlic may be interference with quorum sensing, a common regulatory process between bacterial cells coupling gene expression to cell density as suggested by others (Gonzalez and Keshavan 2006; Bodini *et al.*^2009^; Dessaux, Chapelle and Faure 2011).

The systemic neonicotinoid clothianidin used in our study did not affect the fungal and the prokaryotic soil community structures, both in the pot and in the field experiment. However, the concentration of clothianidin was not monitored and therefore exposure to the compound was not confirmed. It is possible that the insecticide has been partially or completely degraded before effects became manifest although half-life of this chemical in soil is supposed to range between 20 and 1000 weeks (Simon-Delso *et al.*^2015^). Pesticide-degrading microorganisms can reduce the clothianidin concentration as it is degradable aerobically and anaerobically by microbes (Mulligan *et al.*^2016^). Moreover, studies with other systemic neonicotinoids have documented effects on soil fungal and bacterial communities, confirming that clothianidin can potentially have adverse effects on microbial communities (Singh and Singh 2005; Cai *et al.*^2015^, ^2016^; Zaller *et al.*^2016^).

The release of microorganisms to soil for pest control offers great potential and benefits for agriculture. Particularly, entomopathogenic fungi provide an alternative to chemical pesticides or may allow to reduce application of such chemicals and their release to the environment. Registration of entomopathogenic fungi for pest control requires knowledge on possible effects on soil microbial communities. This study showed that *M.brunneum* ART2825 formulated as FCBK and F_cap_, in contrast to the application of fungal spores only, can cause small changes in fungal communities. However, changes were in the same range or even smaller than changes caused by BK (the non-fungal compound of the formulation FCBK), or natural fluctuations in community structures. Amplicon sequencing proved to be a powerful tool for simultaneously assessing exposure to the released strain and effects on the community structure of soil microorganisms. Future investigation should focus on specific functional groups (such as *Rhizobia,* or mycorrhizal fungi) or use meta-proteomics or transcriptomics approaches to assess possible effects at the functional level. This may provide complementary knowledge on the effects of BCAs on microbial communities.

## Supplementary Material

Supplement Files
