# Supplementary material for: Assessing effects of the entomopathogenic fungus Metarhizium brunneum on soil microbial communities in Agriotes spp. biological pest control
Source: FEMS Microbiol Ecol. 2017 Sep 11;93(10):fix117. doi: 10.1093/femsec/fix117 (PMC5812499; doi:10.1093/femsec/fix117)
Supplement: Supplement Files [file fix117_supplement_files.zip › supplemental_figure_captions.docx]

Figure S1: Experimental design in the field experiment. Each plot measured 8.3 times 3 m (4 rows of potato plants). Three plots together formed a block and blocks were separated by a 70 cm wide path (thick line). The 90 plots were surrounded by a buffer zone. Colours indicate different treatments, each replicated six times.

Figure S2: Percentage of damaged potato tubers of 50 potato tubers per plot from each treatment in the field experiment (n = 6).

Figure S3: Rarefaction curve analysis for the fungal (A) and prokaryotic (B) sequencing dataset including the OTU of the applied strain of the pot experiment (n = 6). Colours of curves correspond to sampling time points: week 0, 7 and 15 were coloured green, blue and orange. Relative abundance of all fungal phyla (C) and prokaryotic phyla with an abundance greater than 1 % (D). ^a^ indicates a taxon including all phyla with an abundance of less than 1 %.

Figure S4: OTU richness of fungal (A) and prokaryotic (B) communities per treatment and sampling time point in the pot experiment (n = 6) and OTU richness across the long side of the field of the fungal (C) and the prokaryotic (D) communities (n = 9). Letters (a, b) indicate significant differences among samples of the three time points in each treatment and * indicates significant differences between a treatment and the corresponding untreated control at the respective sampling time point (p ≤ 0.05).

Fig. S5: Relative sequence abundance of prokaryotic OTUs (classified to lowest identified rank) among different treatments and time points (n = 6). * indicates a significant difference between treatments and untreated pots at respective sampling time points (p < 0.05).

Figure S6: Rarefaction curve analysis for the fungal (A) and prokaryotic (B) sequencing dataset including the OTU of the applied strain of the field experiment (n = 6). Colours of curves correspond to sampling time points: week 0, 7 and 15 were coloured green, blue and orange. Relative abundance of all fungal phyla (C) and prokaryotic phyla with an abundance greater than 1 % (D). ^a^ indicates a taxon including all phyla with an abundance of less than 1 %, ^b^ indicates an archaeal phylum.
