# Supplementary material for: Assessing effects of the entomopathogenic fungus Metarhizium brunneum on soil microbial communities in Agriotes spp. biological pest control
Source: FEMS Microbiol Ecol. 2017 Sep 11;93(10):fix117. doi: 10.1093/femsec/fix117 (PMC5812499; doi:10.1093/femsec/fix117)
Supplement: Supplement Files [file fix117_supplement_files.zip › supplemental_tables.docx]

Table S1: Number of isolates identified as *M brunneum* ARSEF 2825 using six SSR markers in the pot and the field experiment at three sampling time points and isolates obtained from infected cadavers during the monitoring of *A. obscurus* larvae retrieved from the pot experiment. In parentheses: number of strains selected for genotyping based on SSR analyses.

|  |  | Number of isolates identified as applied strain | | | |
| --- | --- | --- | --- | --- | --- |
| Experiment | Treatment | Week 0 | Week 7^a^ / 9^b^ | Week 15^a^ / 16^b^ | Infected cadaver |
| Pot | Untreated | 1 (5) | NA | 0 (2) | NC |
| Pot | BK | 0 (5) | NA | NA | NC |
| Pot | Insec | 0 (6) | NA | NA | 1 (1) |
| Pot | FCBK | 0 (6) | 2 (2) | 6 (6) | 1 (1) |
| Pot | F_cap_ | 0 (6) | 2 (2) | 5 (6) | 2 (4) |
| Pot | F_powd_ | 0 (6) | 1 (1) | 6 (6) | 7 (8) |
| Pot | G_cap_ | 0 (6) | NA | NA | NC |
| Pot | FCBK+G_cap_ | 0 (6) | 2 (2) | 6 (6) | 2 (2) |
| Pot | F_cap_+G_cap_ | 2 (6) | 1 (2) | 5 (6) | 2 (3) |
| Field | Untreated | 0 (6) | 0 (6) | 0 (6) | NA |
| Field | F_cap_ | 0 (6) | 0 (6) | 0 (6) | NA |
| Field | F_gran_ | 0 (6) | 2 (6) | 0 (6) | NA |
| Field | FCBK | 0 (6) | 5 (6) | 6 (6) | NA |
| Field | Insec | 0 (6) | 0 (6) | 0 (6) | NA |

NA … not assessed

NC … no infected cadavers

^a^ … sampling time points pot experiment

^b^ … sampling time points field experiment

Table S2: Pairwise ANOSIM comparisons based on Bray Curtis similarities of the fungal and prokaryotic communities in the pot and field experiment among treatments (n=6), among garlic- (n=18) and not-garlic treated pots (n=36), among sampling time points of untreated pots and plots (n=6), among blocks along the long side of the field (n=9).

| Experiment | Organism | Paired groups | ANOSIM R |
| --- | --- | --- | --- |
| Pot | Fungi | BK_7 & Untreated_7 | 0.55*** |
| Pot | Fungi | F_cap_+G_cap__7 & Untreated_7 | -0.006 |
| Pot | Fungi | F_cap__7 & Untreated_7 | 0.415* |
| Pot | Fungi | FCBK_7 & Untreated_7 | 0.506** |
| Pot | Fungi | FCBK+G_cap__7 & Untreated_7 | 0.094 |
| Pot | Fungi | F_powd__7 & Untreated_7 | -0.028 |
| Pot | Fungi | G_cap__7 & Untreated_7 | 0.033 |
| Pot | Fungi | Insec_7 & Untreated_7 | 0.02 |
| Pot | Fungi | BK_15 & Untreated_15 | 0.459* |
| Pot | Fungi | F_cap_+G_cap__15 & Untreated_15 | 0.459** |
| Pot | Fungi | F_cap__15 & Untreated_15 | 0.278** |
| Pot | Fungi | FCBK_15 & Untreated_15 | 0.509** |
| Pot | Fungi | FCBK+G_cap__15 & Untreated_15 | 0.341** |
| Pot | Fungi | F_powd__15 & Untreated_15 | 0.024 |
| Pot | Fungi | G_cap__15 & Untreated_15 | 0.031 |
| Pot | Fungi | Insec_15 & Untreated_15 | -0.07 |
| Pot | Fungi | BK_7 & FCBK_7 | 0.496** |
| Pot | Fungi | BK_7 & F_cap__7 | 0.719** |
| Pot | Fungi | FCBK_7 & F_cap__7 | 0.644** |
| Pot | Fungi | BK_15 & FCBK_15 | 0.433** |
| Pot | Fungi | BK_15 & F_cap_G_cap__15 | 0.557** |
| Pot | Fungi | FCBK_15 & F_cap_G_cap__15 | 0.676** |
| Pot | Fungi | Untreated_0 & Untreated_7 | 0.109 |
| Pot | Fungi | Untreated_0 & Untreated_15 | 0.454** |
| Pot | Fungi | Untreated_7 & Untreated_15 | 0.194* |
| Pot | Fungi | BK_0 & BK_7 | 0.6** |
| Pot | Fungi | BK_0 & BK_15 | 0.42* |
| Pot | Fungi | BK_7 & BK_15 | -0.026 |
| Pot | Fungi | F_cap_G_cap__0 & F_cap_G_cap__7 | 0.319* |
| Pot | Fungi | F_cap_G_cap__0 & F_cap_G_cap__15 | 0.454** |
| Pot | Fungi | F_cap_G_cap__7 & F_cap_G_cap__15 | 0.317* |
| Pot | Fungi | F_cap__0 & F_cap__7 | 0.389* |
| Pot | Fungi | F_cap__0 & F_cap__15 | 0.22* |
| Pot | Fungi | F_cap__7 & F_cap__15 | 0.543* |
| Pot | Fungi | FCBK_0 & FCBK_7 | 0.574** |
| Pot | Fungi | FCBK_0 & FCBK_15 | 0.591** |
| Pot | Fungi | FCBK_7 & FCBK_15 | 0.054 |
| Pot | Prokaryota | BK_7 & Untreated_7 | -0.089 |
| Pot | Prokaryota | F_cap_+G_cap__7 & Untreated_7 | 0.556** |
| Pot | Prokaryota | F_cap__7 & Untreated_7 | 0.196 |
| Pot | Prokaryota | FCBK_7 & Untreated_7 | 0.141* |
| Pot | Prokaryota | FCBK+G_cap__7 & Untreated_7 | 0.819** |
| Pot | Prokaryota | F_powd__7 & Untreated_7 | 0.25* |
| Pot | Prokaryota | G_cap__7 & Untreated_7 | 0.9** |
| Pot | Prokaryota | Insec_7 & Untreated_7 | 0.078 |
| Pot | Prokaryota | BK_15 & Untreated_15 | -0.009 |
| Pot | Prokaryota | F_cap_+G_cap__15 & Untreated_15 | 0.57** |
| Pot | Prokaryota | F_cap__15 & Untreated_15 | 0.063 |
| Pot | Prokaryota | FCBK_15 & Untreated_15 | 0.019 |
| Pot | Prokaryota | FCBK+G_cap__15 & Untreated_15 | 0.6** |
| Pot | Prokaryota | F_powd__15 & Untreated_15 | 0.026 |
| Pot | Prokaryota | G_cap__15 & Untreated_15 | 0.585** |
| Pot | Prokaryota | Insec_15 & Untreated_15 | -0.063 |
| Pot | Prokaryota | F_cap_+G_cap__7 & G_cap__7 | 0.156 |
| Pot | Prokaryota | F_cap_+G_cap__7 & FCBK+G_cap__7 | 0.089 |
| Pot | Prokaryota | G_cap__7 & FCBK+G_cap__7 | 0.07 |
| Pot | Prokaryota | F_cap_+G_cap__15 & G_cap__15 | 0.078 |
| Pot | Prokaryota | F_cap_+G_cap__15 & FCBK+G_cap__15 | -0.087 |
| Pot | Prokaryota | G_cap__15 & FCBK+G_cap__15 | -0.022 |
| Pot | Prokaryota | Untreated_0 & Untreated_7 | 0.646** |
| Pot | Prokaryota | Untreated_0 & Untreated_15 | 0.891** |
| Pot | Prokaryota | Untreated_7 & Untreated_15 | 0.426** |
| Pot | Prokaryota | BK_0 & BK_7 | 0.789** |
| Pot | Prokaryota | BK_0 & BK_15 | 0.974** |
| Pot | Prokaryota | BK_7 & BK_15 | 0.454** |
| Pot | Prokaryota | F_cap_G_cap__0 & F_cap_G_cap__7 | 0.572** |
| Pot | Prokaryota | F_cap_G_cap__0 & F_cap_G_cap__15 | 0.783** |
| Pot | Prokaryota | F_cap_G_cap__7 & F_cap_G_cap__15 | 0.322* |
| Pot | Prokaryota | F_cap__0 & F_cap__7 | 0.65** |
| Pot | Prokaryota | F_cap__0 & F_cap__15 | 0.883** |
| Pot | Prokaryota | F_cap__7 & F_cap__15 | 0.515** |
| Pot | Prokaryota | FCBK_0 & FCBK_7 | 0.239** |
| Pot | Prokaryota | FCBK_0 & FCBK_15 | 0.772** |
| Pot | Prokaryota | FCBK_7 & FCBK_7 | 0.193** |
| Pot | Prokaryota | FCBK+G_cap__0 & FCBK+G_cap__7 | 0.73** |
| Pot | Prokaryota | FCBK+G_cap__0 & FCBK+G_cap__15 | 0.807** |
| Pot | Prokaryota | FCBK+G_cap__7 & FCBK+G_cap__15 | 0.393** |
| Pot | Prokaryota | F_powd__0 & F_powd__7 | 0.606** |
| Pot | Prokaryota | F_powd__0 & F_powd__15 | 0.889** |
| Pot | Prokaryota | F_powd__7 & F_powd__15 | 0.206* |
| Pot | Prokaryota | G_cap__0 & G_cap__7 | 0.706** |
| Pot | Prokaryota | G_cap__0 & G_cap__15 | 0.885** |
| Pot | Prokaryota | G_cap__7 & G_cap__15 | 0.463** |
| Pot | Prokaryota | Insec_0 & Insec_7 | 0.724** |
| Pot | Prokaryota | Insec_0 & Insec_15 | 0.698** |
| Pot | Prokaryota | Insec_7 & Insec_15 | 0.644** |
| Field | Fungi | Untreated_0 & Untreated_9 | 0.112 |
| Field | Fungi | Untreated_0 & Untreated_18 | 0.267** |
| Field | Fungi | Untreated_9 & Untreated_18 | -0.013 |
| Field | Fungi | 0 m & 9 m | -0.026 |
| Field | Fungi | 0 m & 18 m | 0.263*** |
| Field | Fungi | 0 m & 27 m | 0.641*** |
| Field | Fungi | 0 m & 36 m | 0.867*** |
| Field | Fungi | 0 m & 45 m | 0.731*** |
| Field | Fungi | 0 m & 54 m | 0.913*** |
| Field | Fungi | 0 m & 63 m | 0.592*** |
| Field | Fungi | 0 m & 72 m | 0.729*** |
| Field | Fungi | 0 m & 81 m | 0.751*** |
| Field | Fungi | 9 m & 18 m | 0.167* |
| Field | Fungi | 9 m & 27 m | 0.664*** |
| Field | Fungi | 9 m & 36 m | 0.827*** |
| Field | Fungi | 9 m & 45 m | 0.696*** |
| Field | Fungi | 9 m & 54 m | 0.862*** |
| Field | Fungi | 9 m & 63 m | 0.558*** |
| Field | Fungi | 9 m & 72 m | 0.669*** |
| Field | Fungi | 9 m & 81 m | 0.678*** |
| Field | Fungi | 18 m & 27 m | 0.203** |
| Field | Fungi | 18 m & 36 m | 0.592*** |
| Field | Fungi | 18 m & 45 m | 0.428*** |
| Field | Fungi | 18 m & 54 m | 0.664*** |
| Field | Fungi | 18 m & 63 m | 0.523*** |
| Field | Fungi | 18 m & 72 m | 0.623*** |
| Field | Fungi | 18 m & 81 m | 0.614*** |
| Field | Fungi | 27 m & 36 m | 0.24** |
| Field | Fungi | 27 m & 45 m | 0.184** |
| Field | Fungi | 27 m & 54 m | 0.452*** |
| Field | Fungi | 27 m & 63 m | 0.402*** |
| Field | Fungi | 27 m & 72 m | 0.571*** |
| Field | Fungi | 27 m & 81 m | 0.7*** |
| Field | Fungi | 36 m & 45 m | 0.068 |
| Field | Fungi | 36 m & 54 m | 0.398*** |
| Field | Fungi | 36 m & 63 m | 0.482*** |
| Field | Fungi | 36 m & 72 m | 0.703*** |
| Field | Fungi | 36 m & 81 m | 0.769*** |
| Field | Fungi | 45 m & 54 m | 0.336*** |
| Field | Fungi | 45 m & 63 m | 0.418*** |
| Field | Fungi | 45 m & 72 m | 0.572*** |
| Field | Fungi | 45 m & 81 m | 0.623*** |
| Field | Fungi | 54 m & 63 m | 0.308*** |
| Field | Fungi | 54 m & 72 m | 0.557*** |
| Field | Fungi | 54 m & 81 m | 0.641*** |
| Field | Fungi | 62 m & 72 m | 0.079 |
| Field | Fungi | 62 m & 81 m | 0.389*** |
| Field | Fungi | 72 m & 81 m | 0.235** |
| Field | Prokaryota | 0 m & 9 m | -0.011 |
| Field | Prokaryota | 0 m & 18 m | 0.16** |
| Field | Prokaryota | 0 m & 27 m | 0.795*** |
| Field | Prokaryota | 0 m & 36 m | 0.967*** |
| Field | Prokaryota | 0 m & 45 m | 0.996*** |
| Field | Prokaryota | 0 m & 54 m | 0.939*** |
| Field | Prokaryota | 0 m & 63 m | 0.854*** |
| Field | Prokaryota | 0 m & 72 m | 0.886*** |
| Field | Prokaryota | 0 m & 81 m | 0.856*** |
| Field | Prokaryota | 9 m & 18 m | 0.115* |
| Field | Prokaryota | 9 m & 27 m | 0.783*** |
| Field | Prokaryota | 9 m & 36 m | 0.965*** |
| Field | Prokaryota | 9 m & 45 m | 0.997*** |
| Field | Prokaryota | 9 m & 54 m | 0.924*** |
| Field | Prokaryota | 9 m & 63 m | 0.799*** |
| Field | Prokaryota | 9 m & 72 m | 0.863*** |
| Field | Prokaryota | 9 m & 81 m | 0.798*** |
| Field | Prokaryota | 18 m & 27 m | 0.334** |
| Field | Prokaryota | 18 m & 36 m | 0.855*** |
| Field | Prokaryota | 18 m & 45 m | 0.942*** |
| Field | Prokaryota | 18 m & 54 m | 0.671*** |
| Field | Prokaryota | 18 m & 63 m | 0.334*** |
| Field | Prokaryota | 18 m & 72 m | 0.499*** |
| Field | Prokaryota | 18 m & 81 m | 0.544*** |
| Field | Prokaryota | 27 m & 36 m | 0.47*** |
| Field | Prokaryota | 27 m & 45 m | 0.55*** |
| Field | Prokaryota | 27 m & 54 m | 0.22* |
| Field | Prokaryota | 27 m & 63 m | 0.487*** |
| Field | Prokaryota | 27 m & 72 m | 0.859*** |
| Field | Prokaryota | 27 m & 81 m | 0.92*** |
| Field | Prokaryota | 36 m & 45 m | -0.038 |
| Field | Prokaryota | 36 m & 54 m | 0.419*** |
| Field | Prokaryota | 36 m & 63 m | 0.854*** |
| Field | Prokaryota | 36 m & 72 m | 0.969*** |
| Field | Prokaryota | 36 m & 81 m | 0.995*** |
| Field | Prokaryota | 45 m & 54 m | 0.38*** |
| Field | Prokaryota | 45 m & 63 m | 0.907*** |
| Field | Prokaryota | 45 m & 72 m | 0.995*** |
| Field | Prokaryota | 45 m & 81 m | 1*** |
| Field | Prokaryota | 54 m & 63 m | 0.49*** |
| Field | Prokaryota | 54 m & 72 m | 0.847*** |
| Field | Prokaryota | 54 m & 81 m | 0.933*** |
| Field | Prokaryota | 62 m & 72 m | 0.354*** |
| Field | Prokaryota | 62 m & 81 m | 0.68*** |
| Field | Prokaryota | 72 m & 81 m | 0.219** |

* p ≤ 0.05

** p ≤ 0.01

*** p ≤ 0.001

Table S3: Effects of treatments, garlic, time and interactions on fungal and prokaryotic communities in soil of the pot experiment based on BC dissimilarities assessed with overall PERMANOVA corrected p-values of pairwise PERMANOVA tests among untreated pots and treatments.

| Test | Organism | Factor | Degrees of freedom | Sums of squares | Mean of sums of squares | Pseudo F-statistic | R^2^ | P-value |  |  |
| --- | --- | --- | --- | --- | --- | --- | --- | --- | --- | --- |
| Overall | Fungi | Treatment | 8 | 2.2011 | 0.2751 | 2.7359 | 0.1196 | 0.0001 |  |  |
|  |  | Time | 1 | 0.6750 | 0.6750 | 6.7116 | 0.0367 | 0.0001 |  |  |
|  |  | Treatmentxtime | 7 | 1.0440 | 0.1305 | 1.2977 | 0.0567 | 0.0013 |  |  |
|  |  | Residuals | 143 | 14.4817 | 0.1006 | NA | 0.7870 | NA |  |  |
|  |  | Total | 161 | 18.4017 | NA | NA | 1.0000 | NA |  |  |
|  | Prokaryota | Treatment | 8 | 0.4099 | 0.0512 | 1.3207 | 0.0617 | 0.0001 |  |  |
|  |  | Time | 1 | 0.2873 | 0.2873 | 7.4064 | 0.0433 | 0.0001 |  |  |
|  |  | Treatmentxtime | 7 | 0.3555 | 0.0444 | 1.1456 | 0.0536 | 0.0001 |  |  |
|  |  | Residuals | 143 | 5.5863 | 0.0388 | NA | 0.8414 | NA |  |  |
|  |  | Total | 161 | 6.6391 | NA | NA | 1.0000 | NA |  |  |
|  |  |  |  |  |  |  |  |  |  |  |
|  |  | Sampling time | Pairwise comparison of each treatment with untreated (p-value) | | | | | | | |
|  |  |  | BK | F+G_cap_ | F_cap_ | FCBK | FCBK+G_cap_ | F_powd_ | G_cap_ | Insec |
| Pairwise | Fungi | Week 0 | 0.5240 | 0.1116 | 0.5157 | 0.3128 | 0.1116 | 0.3698 | 0.3128 | 0.3128 |
|  |  | Week 7 | 0.0061 | 0.5173 | 0.0221 | 0.0061 | 0.1632 | 0.8854 | 0.3857 | 0.0809 |
|  |  | Week 15 | 0.0161 | 0.0060 | 0.0085 | 0.0060 | 0.0085 | 0.1330 | 0.2889 | 0.7119 |
|  | Prokaryota | Week 0 | 0.0801 | 0.0666 | 0.2473 | 0.5454 | 0.1671 | 0.3876 | 0.1868 | 0.0801 |
|  |  | Week 7 | 0.7175 | 0.0074 | 0.0318 | 0.0318 | 0.0074 | 0.0420 | 0.0074 | 0.0729 |
|  |  | Week 15 | 0.6474 | 0.0063 | 0.3710 | 0.4478 | 0.0063 | 0.3710 | 0.0063 | 0.2771 |

Table S4: Percent contribution of the 100 most abundant fungal OTUs to differences in BC dissimilarities between a treatment and the respective control at week 7 and 15 analysed using SIMPER, and the taxonomic classification of each OTU. Treatments were selected based on a significant pairwise ANOSIM and PERMANOVA value assessing fungal community structures among treatments.

| Percent contribution (SIMPER) % | | | | | | | | | | | |
| --- | --- | --- | --- | --- | --- | --- | --- | --- | --- | --- | --- |
| Week 7 | | | | | | Week 15 | | | | | |
| OTU | Taxonomic classification | BK | FCBK | F_cap_ | OTU | Taxonomic classification | BK | FCBK | F_cap_ | FCBK+G_cap_ | F_cap_+G_cap_ |
| OTU 1* | Bionectriaceae | 1.49 | 12.31 | 0.7 | OTU 1* | Bionectriaceae | 0.93 | 10.55 | 0.47 | 2.01 | 1.02 |
| OTU 100 | Mortierellales | 0.9 | 1.14 | 1.25 | OTU 100 | Mortierellales | 0.87 | 0.65 | 0.61 | 0.9 | 0.93 |
| OTU 102 | Sordariales | 0.74 | 0.63 | 0.71 | OTU 103 | Agaricales | 0.66 | 0.62 | 0.67 | 0.78 | 0.64 |
| OTU 104 | Cladorrhinum | 0.72 | 0.87 | 0.76 | OTU 106 | Nectriaceae | 0.92 | 0.49 | 0.59 | 0.86 | 1.34 |
| OTU 106 | Nectriaceae | 0.38 | 0.55 | 0.69 | OTU 11* | Rhizopus oryzae | 9.62 | 0.85 | 0.81 | 1.05 | 0.82 |
| OTU 107 | Pseudeurotium | 0.44 | 0.68 | 0.64 | OTU 114 | Nectriaceae | 1.1 | 1.16 | 0.93 | 0.86 | 1.01 |
| OTU 11* | Rhizopus oryzae | 12.03 | 2.74 | 0.9 | OTU 121 | Ascomycota | 0.35 | 0.37 | 0.89 | 2.45 | 0.66 |
| OTU 114 | Nectriaceae | 0.52 | 2.47 | 0.4 | OTU 123 | Agaricales | 0.92 | 0.76 | 0.8 | 0.51 | 1.53 |
| OTU 123 | Agaricales | 0.76 | 1.26 | 0.97 | OTU 125 | Volutella ciliata | 0.31 | 0.39 | 1.49 | 0.35 | 0.53 |
| OTU 127 | Talaromyces variabilis | 0.08 | 0.16 | 0.07 | OTU 1254 | Colletotrichum dematium | 1.73 | 2.01 | 2.24 | 1.75 | 2.05 |
| OTU 13* | Nectriaceae | 0.92 | 0.8 | 1.18 | OTU 13* | Nectriaceae | 1.48 | 1.13 | 0.75 | 2.81 | 0.5 |
| OTU 140 | Trichoderma | 0.42 | 0.41 | 0.69 | OTU 132 | Sordariomycetes | 0.72 | 1.08 | 1 | 0.82 | 0.94 |
| OTU 142 | Gaeumannomyces graminis | 0.12 | 0.12 | 0.13 | OTU 135 | Aspergillus fumigatus | 0.33 | 0.3 | 0.41 | 0.64 | 0.33 |
| OTU 144 | Pezizaceae | 1.49 | 1.61 | 3.21 | OTU 144 | Pezizaceae | 1.72 | 1.79 | 1.54 | 2.53 | 2.44 |
| OTU 15 | Sordariomycetes WF149 | 2.03 | 2.33 | 2.58 | OTU 147 | Helotiales | 0.51 | 1.04 | 0.53 | 0.57 | 0.46 |
| OTU 154 | Actinomucor elegans | 0.46 | 0.45 | 0.46 | OTU 148 | Torula | 0.26 | 0.3 | 0.4 | 0.32 | 0.94 |
| OTU 16 | Davidiellaceae | 1.34 | 1.43 | 1.25 | OTU 15 | Sordariomycetes WF149 | 1.72 | 1.62 | 1.83 | 2.25 | 2.36 |
| OTU 166 | Hypocreales | 1.02 | 1.19 | 1.15 | OTU 154 | Actinomucor elegans | 0.8 | 0.44 | 0.39 | 1.18 | 0.38 |
| OTU 17 | Tetracladium | 0.86 | 0.77 | 1.44 | OTU 16 | Davidiellaceae | 0.61 | 0.77 | 1.06 | 0.57 | 0.8 |
| OTU 171 | Myrothecium verrucaria | 0.73 | 0.7 | 0.84 | OTU 17 | Tetracladium | 0.82 | 0.81 | 0.65 | 0.99 | 0.84 |
| OTU 173 | Gibberella zeae | 0.7 | 0.64 | 0.8 | OTU 171 | Myrothecium verrucaria | 0.96 | 0.58 | 0.8 | 0.81 | 0.62 |
| OTU 178 | Sordariales | 0.47 | 0.74 | 0.7 | OTU 18 | Acremonium rutilum | 1.59 | 1.78 | 2.11 | 1.56 | 1.38 |
| OTU 18 | Acremonium rutilum | 1.86 | 2.18 | 1.98 | OTU 1889 | Fungi | 1.23 | 1.29 | 1.38 | 1.22 | 1.66 |
| OTU 1811 | Mortierella elongata | 0.47 | 0.47 | 0.53 | OTU 189 | Lasiosphaeriaceae | 1.81 | 1.94 | 2.44 | 1.87 | 2.13 |
| OTU 19 | Trichocladium asperum | 1.47 | 2.02 | 1.51 | OTU 19 | Trichocladium asperum | 1.35 | 1.64 | 1.81 | 2.4 | 1.55 |
| OTU 197 | Dothideomycetes WF116 | 0.15 | 0.16 | 1.57 | OTU 197 | Dothideomycetes WF116 | 0.12 | 0.16 | 0.18 | 0.19 | 0.15 |
| OTU 198 | Sordariomycetes | 0.3 | 0.45 | 0.95 | OTU 199 | Lasiosphaeriaceae | 0.86 | 0.84 | 1.16 | 0.76 | 1 |
| OTU 2 | Sordariomycetes | 2.86 | 3.82 | 5.98 | OTU 2 | Sordariomycetes | 1.37 | 2.12 | 1.75 | 1.34 | 0.9 |
| OTU 20 | Mortierella elongata | 0.93 | 1.16 | 1.26 | OTU 20 | Mortierella elongata | 1.12 | 1.02 | 1.13 | 1.26 | 1.37 |
| OTU 2046 | Sordariales | 0.56 | 1.2 | 0.68 | OTU 200 | Penicillium olsonii | 0.92 | 0.96 | 1.12 | 1.07 | 1.44 |
| OTU 21 | Talaromyces | 6.86 | 0.75 | 0.92 | OTU 204 | Ascomycota | 0.47 | 0.94 | 1.12 | 1.17 | 0.82 |
| OTU 2114 | Podospora | 0.21 | 0.19 | 0.2 | OTU 2046 | Sordariales | 0.59 | 0.57 | 0.5 | 1.24 | 0.51 |
| OTU 25 | Fusarium merismoides | 0.97 | 0.72 | 0.74 | OTU 205 | Trechisporales | 0.94 | 0.91 | 1.21 | 1.04 | 1.21 |
| OTU 260 | Psathyrella stercoraria | 0.28 | 0.25 | 0.22 | OTU 21 | Talaromyces | 6.64 | 4.32 | 0.44 | 0.7 | 0.57 |
| OTU 262 | Acremonium aff curvulum NRRL 62959 | 0.49 | 0.49 | 0.7 | OTU 215 | Fungi | 0.65 | 0.85 | 1.6 | 0.76 | 0.87 |
| OTU 2683 | Cadophora luteo olivacea | 0.95 | 1 | 1.12 | OTU 23 | Ilyonectria torresensis | 0.59 | 0.63 | 1.12 | 0.66 | 1.27 |
| OTU 27 | uncultured Coprinellus | 0.9 | 0.82 | 1.13 | OTU 2364 | Podospora | 0.81 | 0.89 | 0.92 | 0.58 | 1.54 |
| OTU 272 | Fungi | 0.55 | 0.84 | 0.37 | OTU 239 | Spizellomycetales | 0.35 | 0.34 | 0.34 | 0.79 | 0.74 |
| OTU 29 | Ascomycota | 0.81 | 0.75 | 1.4 | OTU 25 | Fusarium merismoides | 0.89 | 0.99 | 0.95 | 0.82 | 0.92 |
| OTU 30 | Trichoderma gamsii | 1.42 | 1.14 | 1.23 | OTU 27 | uncultured Coprinellus | 0.8 | 0.7 | 0.6 | 0.61 | 0.95 |
| OTU 305 | Lasiosphaeriaceae | 0.85 | 0.29 | 0.24 | OTU 272 | Fungi | 0.99 | 0.63 | 0.51 | 1.38 | 1.71 |
| OTU 31 | uncultured Tetracladium | 0.98 | 0.89 | 1.18 | OTU 286 | Thanatephorus cucumeris | 1.05 | 1.07 | 1.48 | 1.24 | 1.35 |
| OTU 313 | Chaetothyriales | 0.41 | 0.27 | 0.44 | OTU 29 | Ascomycota | 1.15 | 0.9 | 0.98 | 0.96 | 1.63 |
| OTU 32 | Ascomycota | 1.26 | 1.6 | 2.32 | OTU 295 | Piloderma | 0 | 0 | 0 | 0 | 0 |
| OTU 320 | uncultured Calyptella | 0 | 1.18 | 0.09 | OTU 30 | Trichoderma gamsii | 0.47 | 0.61 | 0.61 | 3.48 | 0.53 |
| OTU 327 | Lasiosphaeriaceae | 0.54 | 0.34 | 0.28 | OTU 31 | uncultured Tetracladium | 0.66 | 0.81 | 1 | 0.8 | 0.98 |
| OTU 358 | Fungi | 0 | 0.33 | 0 | OTU 32 | Ascomycota | 0.47 | 0.55 | 1 | 0.95 | 0.8 |
| OTU 36 | Pseudeurotium | 1.26 | 1.06 | 0.97 | OTU 338 | Saccharomycete P20 | 0.28 | 0.26 | 0.21 | 0.35 | 0.55 |
| OTU 365 | Tirispora | 0.84 | 0.32 | 0.54 | OTU 343 | Cordyceps | 0.35 | 0.36 | 1.02 | 0.32 | 0.47 |
| OTU 37 | Myrothecium cinctum | 1.24 | 1.98 | 2.41 | OTU 3464 | Sordariomycetes | 0.51 | 0.6 | 0.58 | 0.49 | 0.47 |
| OTU 38 | Chaetomiaceae | 1.12 | 1.16 | 1.69 | OTU 36 | Pseudeurotium | 0.92 | 0.73 | 1.09 | 0.71 | 1.17 |
| OTU 3918 | Beauveria | 0 | 0.14 | 0 | OTU 37 | Myrothecium cinctum | 0.75 | 0.9 | 0.99 | 0.97 | 0.98 |
| OTU 395 | Arthrobotrys musiformis | 0.25 | 0.46 | 0.29 | OTU 379 | Cephalotrichum microsporum | 0.19 | 0.14 | 0.25 | 0.29 | 0.39 |
| OTU 4 | Monographella cucumerina | 2.7 | 2.89 | 3.91 | OTU 38 | Chaetomiaceae | 0.6 | 0.69 | 0.56 | 0.52 | 0.46 |
| OTU 41 | Lasiosphaeriaceae | 1.57 | 0.5 | 0.61 | OTU 384 | Helotiales | 1.03 | 1.1 | 1.21 | 1.11 | 1.28 |
| OTU 42 | Pezizomycetes | 0.53 | 0.59 | 0.98 | OTU 4 | Monographella cucumerina | 2.09 | 1.52 | 1.76 | 1.88 | 1.57 |
| OTU 43 | Lasiosphaeriaceae | 1.13 | 0.74 | 3.22 | OTU 40 | Cladosporium sphaerospermum | 1.55 | 1.64 | 1.99 | 1.57 | 2.6 |
| OTU 439 | Ascomycota | 0.62 | 0.48 | 0.57 | OTU 407 | Agaricomycetes | 0.55 | 0.53 | 0 | 0 | 0.04 |
| OTU 44 | Neonectria ramulariae | 0.88 | 0.81 | 0.77 | OTU 41 | Lasiosphaeriaceae | 1.41 | 1.52 | 1.87 | 1.49 | 1.63 |
| OTU 45* | Mortierella | 0.82 | 1.09 | 1.51 | OTU 42 | Pezizomycetes | 0.86 | 0.98 | 1.97 | 0.75 | 1.02 |
| OTU 4553 | Trichosporon dulcitum | 0.69 | 0.61 | 0.71 | OTU 43 | Lasiosphaeriaceae | 1.58 | 1.47 | 1.8 | 1.3 | 1.66 |
| OTU 4577 | Hypocreales | 0.36 | 1.69 | 0.46 | OTU 431 | Elaphomyces muricatus | 0 | 0 | 0 | 0 | 0 |
| OTU 47 | Dipodascaceae | 0.62 | 0.65 | 1.42 | OTU 438 | Fungi | 0.6 | 0.54 | 0.58 | 0.52 | 0.6 |
| OTU 48 | Staphylotrichum coccosporum | 1.06 | 1.13 | 1.6 | OTU 439 | Ascomycota | 0.58 | 0.61 | 0.79 | 0.56 | 0.95 |
| OTU 49 | Myrmecridium | 1.02 | 0.7 | 0.76 | OTU 44 | Neonectria ramulariae | 0.47 | 0.55 | 1.01 | 0.99 | 0.6 |
| OTU 5 | Gibberella intricans | 1.83 | 1.02 | 1.35 | OTU 444 | Arthrinium | 0.57 | 0.61 | 0.9 | 0.56 | 0.67 |
| OTU 51 | Trichosporon scarabaeorum | 0.6 | 1.23 | 0.74 | OTU 45* | Mortierella | 1.11 | 0.81 | 0.79 | 1.03 | 0.95 |
| OTU 524 | Mycena | 0 | 0 | 0 | OTU 4553 | Trichosporon dulcitum | 1.03 | 1.46 | 1.4 | 1.02 | 1.08 |
| OTU 53 | Pyrenochaeta inflorescentiae | 0.6 | 0.6 | 0.71 | OTU 46 | Arthopyreniaceae 2 DoF13 | 0.45 | 0.45 | 1.19 | 0.33 | 0.58 |
| OTU 55 | Neosetophoma samarorum | 0.77 | 0.65 | 0.7 | OTU 469 | Trichocomaceae | 0.82 | 1.03 | 1.01 | 0.82 | 0.72 |
| OTU 56 | Pleosporales | 0.76 | 0.69 | 0.79 | OTU 47 | Dipodascaceae | 0.54 | 0.44 | 0.8 | 1.6 | 0.81 |
| OTU 59 | Pyrenochaeta | 0.63 | 0.58 | 0.7 | OTU 48 | Staphylotrichum coccosporum | 0.61 | 0.73 | 0.63 | 0.79 | 0.66 |
| OTU 6 | Fusarium | 1.94 | 1.66 | 1.72 | OTU 480 | Ophiosphaerella | 0.7 | 0.18 | 0.17 | 0.14 | 0.34 |
| OTU 60 | Cylindrocarpon FKI 4602 | 0.6 | 0.86 | 1.53 | OTU 482 | Nectriaceae | 0.87 | 0.82 | 0.94 | 0.87 | 0.93 |
| OTU 61 | Hypocreales | 0.68 | 1.4 | 1.18 | OTU 485 | Saccharomycete P20 | 0.19 | 0.1 | 0.18 | 0.38 | 0.32 |
| OTU 62 | Peyronellaea glomerata | 1.07 | 0.77 | 1.08 | OTU 486 | Orbiliaceae | 0.57 | 0.97 | 1.15 | 0.42 | 0.49 |
| OTU 624 | Helotiales | 0.53 | 0.56 | 0.6 | OTU 49 | Myrmecridium | 0.74 | 0.67 | 0.65 | 0.49 | 0.76 |
| OTU 627 | Fungi | 0.06 | 0 | 0.81 | OTU 5 | Gibberella intricans | 1.74 | 1.68 | 1.74 | 1.32 | 1.8 |
| OTU 63 | Monographella cucumerina | 1.05 | 1.34 | 1.87 | OTU 51 | Trichosporon scarabaeorum | 0.93 | 0.85 | 0.91 | 0.87 | 0.96 |
| OTU 64 | Mortierellaceae | 0.91 | 0.99 | 0.9 | OTU 53 | Pyrenochaeta inflorescentiae | 1.03 | 0.88 | 1.35 | 1.08 | 1.15 |
| OTU 65 | Chaetomium madrasense | 0.67 | 0.63 | 0.54 | OTU 56 | Pleosporales | 0.54 | 0.64 | 0.78 | 0.55 | 0.48 |
| OTU 676 | Lecanoromycetes | 0.5 | 0 | 0 | OTU 586 | Sordariomycetes | 0 | 0.19 | 0.09 | 0 | 0.2 |
| OTU 68 | Trichosporon laibachii | 1.1 | 1.06 | 1.19 | OTU 59 | Pyrenochaeta | 0.97 | 0.85 | 1.2 | 0.59 | 1.4 |
| OTU 69 | uncultured Tetracladium | 0.5 | 0.33 | 0.77 | OTU 593 | Halosphaeriaceae | 0.76 | 0.7 | 0.85 | 0.64 | 0.81 |
| OTU 700 | Lecanicillium saksenae | 0.24 | 0.24 | 0.26 | OTU 6 | Fusarium | 1.55 | 0.83 | 1.33 | 1.23 | 3.39 |
| OTU 738 | Arthrobotrys | 0.03 | 0 | 0.07 | OTU 60 | Cylindrocarpon FKI 4602 | 0.46 | 0.4 | 0.54 | 0.74 | 0.66 |
| OTU 74 | Cercophora | 1.4 | 1.2 | 1.39 | OTU 61 | Hypocreales | 1.08 | 1.15 | 1.16 | 1.17 | 0.99 |
| OTU 758 | Ascomycota | 0 | 0 | 0.17 | OTU 62 | Peyronellaea glomerata | 0.87 | 0.61 | 0.76 | 0.96 | 0.63 |
| OTU 769 | Fungi | 0.28 | 0.63 | 0.13 | OTU 65 | Chaetomium madrasense | 0.49 | 0.7 | 0.67 | 0.6 | 0.66 |
| OTU 77 | Schizothecium carpinicola | 1.32 | 1.35 | 1.46 | OTU 658 | Hypocreales | 0.38 | 0.37 | 0.47 | 0.34 | 0.44 |
| OTU 79 | Paraphoma chrysanthemicola | 0.52 | 0.74 | 0.84 | OTU 675 | Fungi | 0 | 0.67 | 0 | 0.2 | 0 |
| OTU 8 | Chaetomiaceae | 1.52 | 1.44 | 1.57 | OTU 68 | Trichosporon laibachii | 0.94 | 1.13 | 1.23 | 1.92 | 1.17 |
| OTU 82 | Candida sake | 0.68 | 0.69 | 0.58 | OTU 77 | Schizothecium carpinicola | 1.24 | 1.4 | 1.63 | 1.49 | 1.64 |
| OTU 847 | Phaeosphaeriaceae | 0 | 0 | 0 | OTU 8 | Chaetomiaceae | 5.59 | 5.96 | 6.56 | 5.32 | 5.99 |
| OTU 86 | Alternaria tenuissima | 0.95 | 0.88 | 1.24 | OTU 91 | Tetracladium furcatum | 0.47 | 0.48 | 0.81 | 0.79 | 0.6 |
| OTU 90 | Mortierellales | 0.53 | 0.71 | 0.62 | OTU 92 | Nectriaceae | 0.44 | 0.27 | 0.59 | 0.42 | 0.61 |
| OTU 91 | Tetracladium furcatum | 0.82 | 0.47 | 0.42 | OTU 94 | Myrothecium roridum | 0.56 | 0.58 | 0.87 | 0.56 | 0.56 |
| OTU 94 | Myrothecium roridum | 0.85 | 1.29 | 1.59 | OTU 95 | Alternaria | 0.45 | 0.57 | 0.82 | 0.65 | 0.43 |
| OTU 96 | Acremonium persicinum | 0.93 | 1.17 | 0.97 | OTU 97 | Fungi | 0.5 | 0.62 | 0.8 | 1.36 | 0.64 |
| OTU 98 | Fusarium tricinctum | 3.7 | 0.55 | 0.53 | OTU 99 | Microdochium | 1.27 | 0.91 | 1.01 | 1.08 | 0.96 |

*… OTUs with significant overall PERMANOVA of relative sequence abundance among treatments

Table S5: Percent contribution of the 100 most abundant prokaryotic OTUs to differences in BC dissimilarities between a treatment and the respective control at week 7 and 15 analysed using SIMPER, and the taxonomic classification of each OTU. Treatments were selected based on a significant pairwise ANOSIM and PERMANOVA value assessing fungal community structures among treatments.

| Percent contribution (SIMPER) % | | | | | | | | | |
| --- | --- | --- | --- | --- | --- | --- | --- | --- | --- |
| Week 7 | | | | | Week 15 | | | | |
| OTU | Taxonomic classification | Gcap | FCBK+Gcap | F+Gcap | OTU | Taxonomic classification | Gcap | FCBK+Gcap | F+Gcap |
| OTU 1 | Hyphomicrobiaceae | 0.98 | 0.86 | 0.62 | OTU 1 | Hyphomicrobiaceae | 1.89 | 1.59 | 1.38 |
| OTU 10 | [Chthoniobacteraceae] DA101 | 1.02 | 1.15 | 1.15 | OTU 10 | [Chthoniobacteraceae] DA101 | 1.12 | 0.94 | 1.05 |
| OTU 100 | Kaistobacter | 1.21 | 0.58 | 0.77 | OTU 100 | Kaistobacter | 1.02 | 0.82 | 0.73 |
| OTU 1003 | Verrucomicrobiaceae | 1.07 | 1.13 | 1.32 | OTU 103 | Solirubrobacterales | 0.52 | 0.53 | 0.59 |
| OTU 103 | Solirubrobacterales | 0.6 | 0.67 | 0.59 | OTU 105 | Thermomonas | 1.06 | 0.92 | 1.08 |
| OTU 105 | Thermomonas | 1.11 | 0.8 | 1.09 | OTU 106 | Skermanella | 1.21 | 0.99 | 0.94 |
| OTU 106 | Skermanella | 0.65 | 0.71 | 0.68 | OTU 107 | Anaerolinaceae | 0.76 | 1.65 | 0.62 |
| OTU 107 | Anaerolinaceae | 0.67 | 0.55 | 0.84 | OTU 109* | Acidobacteria-6 iii1-15 | 0.75 | 0.68 | 1.26 |
| OTU 109* | Acidobacteria-6 iii1-15 | 1.12 | 0.96 | 0.88 | OTU 11 | Rhodoplanes | 1.32 | 0.7 | 0.93 |
| OTU 11 | Rhodoplanes | 0.97 | 1 | 0.66 | OTU 110 | Chloroflexi Gitt-GS-136 | 0.79 | 0.57 | 0.81 |
| OTU 110 | Chloroflexi Gitt-GS-136 | 0.72 | 0.79 | 0.88 | OTU 1110 | Cyanobacteria | 0.19 | 0.45 | 0.18 |
| OTU 119 | Acidobacteria-6 iii1-15 | 1.24 | 0.37 | 0.43 | OTU 117 | Chloroflexi Ellin6529 | 0.94 | 0.8 | 0.55 |
| OTU 1191 | Verrucomicrobiaceae | 0.72 | 2.26 | 0.8 | OTU 119 | Acidobacteria-6 iii1-15 | 0.86 | 0.68 | 0.62 |
| OTU 12 | Nitrospira | 0.67 | 0.68 | 0.57 | OTU 12 | Nitrospira | 0.64 | 0.67 | 0.62 |
| OTU 1222 | Agrobacterium | 0.77 | 1.21 | 0.89 | OTU 13 | Acidobacteria-6 iii1-15 | 0.93 | 0.9 | 1.07 |
| OTU 13 | Acidobacteria-6 iii1-15 | 1.15 | 0.88 | 0.78 | OTU 131 | Betaproteobacteria MND1 | 0.82 | 0.84 | 0.93 |
| OTU 131 | Betaproteobacteria MND1 | 1.36 | 1.52 | 0.99 | OTU 137 | Caldilinea | 1.05 | 0.72 | 0.78 |
| OTU 137 | Caldilinea | 1.2 | 1.34 | 1.27 | OTU 1389* | Xanthomonadaceae | 3.33 | 2.47 | 3.51 |
| OTU 14 | Pedomicrobium | 0.93 | 0.94 | 1.04 | OTU 14 | Pedomicrobium | 0.77 | 0.81 | 0.77 |
| OTU 143 | [Chloracidobacteria] PK29 | 0.91 | 0.59 | 0.64 | OTU 148 | Gaiellaceae | 0.89 | 0.64 | 0.72 |
| OTU 148 | Gaiellaceae | 0.63 | 0.66 | 0.68 | OTU 15 | CandidatusNitrososphaera SCA1170 | 0.89 | 1.08 | 0.79 |
| OTU 15 | CandidatusNitrososphaera SCA1170 | 0.93 | 0.72 | 0.53 | OTU 151 | Acidobacteria-6 iii1-15 | 0.79 | 0.95 | 0.78 |
| OTU 1501 | [Pedosphaerales] auto67_4W | 0.97 | 0.76 | 1.04 | OTU 156 | Acidobacteria-6 iii1-15 | 1.2 | 0.97 | 1.13 |
| OTU 156 | Acidobacteria-6 iii1-15 | 0.91 | 0.92 | 1.12 | OTU 159 | Anaerolinea | 0.58 | 1.46 | 0.74 |
| OTU 16 | Geodermatophilaceae | 0.6 | 0.58 | 0.78 | OTU 16 | Geodermatophilaceae | 0.54 | 0.53 | 0.42 |
| OTU 178 | Anaerolineae S0208 | 0.74 | 0.75 | 0.66 | OTU 178 | Anaerolineae S0208 | 0.68 | 0.63 | 0.58 |
| OTU 181 | Solirubrobacterales | 1.41 | 1.52 | 1.21 | OTU 180 | Sinobacteraceae | 0.97 | 1.04 | 0.86 |
| OTU 185 | Mesorhizobium | 1.01 | 0.92 | 1.13 | OTU 181 | Solirubrobacterales | 0.76 | 0.8 | 0.96 |
| OTU 19 | Gaiellaceae | 0.75 | 0.67 | 0.77 | OTU 185 | Mesorhizobium | 0.87 | 0.68 | 0.7 |
| OTU 195 | Chitinophagaceae | 0.75 | 0.65 | 0.92 | OTU 19 | Gaiellaceae | 0.92 | 0.65 | 0.67 |
| OTU 2* | Micrococcaceae | 0.81 | 0.85 | 1.01 | OTU 195 | Chitinophagaceae | 1.01 | 0.73 | 1.05 |
| OTU 20 | Chloroflexi Gitt-GS-136 | 0.51 | 0.6 | 0.75 | OTU 2* | Micrococcaceae | 1.22 | 0.66 | 1.1 |
| OTU 21 | Streptomyces mirabilis | 0.75 | 0.74 | 1.07 | OTU 20 | Chloroflexi Gitt-GS-136 | 0.92 | 0.95 | 1.24 |
| OTU 22 | Anaerolinea | 1.13 | 1.22 | 0.97 | OTU 21 | Streptomyces mirabilis | 1.23 | 1 | 0.73 |
| OTU 228 | Acidimicrobiales C111 | 0.79 | 0.84 | 1.02 | OTU 22 | Anaerolinea | 0.85 | 1.99 | 0.65 |
| OTU 23 | Pedomicrobium | 0.74 | 0.73 | 0.92 | OTU 222 | Phormidium | 2.1 | 2.2 | 3.47 |
| OTU 24 | Gaiellaceae | 0.32 | 0.4 | 0.78 | OTU 2230 | Opitutus | 0.45 | 0.25 | 2.01 |
| OTU 2475 | Luteolibacter | 1.06 | 1.33 | 1.21 | OTU 228 | Acidimicrobiales C111 | 0.72 | 0.79 | 0.78 |
| OTU 25 | Acidobacteria-6 iii1-15 | 1.29 | 0.98 | 0.87 | OTU 23 | Pedomicrobium | 0.79 | 0.62 | 0.95 |
| OTU 27 | Anaerolineae GCA004 | 1.06 | 0.97 | 0.83 | OTU 24 | Gaiellaceae | 0.87 | 0.77 | 0.75 |
| OTU 271* | Acidobacteria-6 iii1-15 | 1.19 | 1.42 | 1.28 | OTU 25 | Acidobacteria-6 iii1-15 | 1.29 | 1.6 | 1.51 |
| OTU 28 | Gaiellaceae | 0.76 | 0.86 | 0.78 | OTU 2564 | Paenibacillus | 0.87 | 0.92 | 0.72 |
| OTU 286 | Anaerolinea | 1.17 | 1.27 | 0.75 | OTU 27 | Anaerolineae GCA004 | 0.76 | 1.38 | 0.83 |
| OTU 29 | Nocardioidaceae | 1.01 | 1.15 | 1.04 | OTU 271* | Acidobacteria-6 iii1-15 | 0.83 | 1.39 | 1.37 |
| OTU 3 | CandidatusXiphinematobacter | 1.42 | 1.65 | 1.43 | OTU 28 | Gaiellaceae | 0.85 | 0.8 | 0.83 |
| OTU 30 | Actinobacteria MB-A2-108 | 0.39 | 0.47 | 0.59 | OTU 286 | Anaerolinea | 0.91 | 1.86 | 1.27 |
| OTU 308 | Nostocaceae | 2.26 | 2.02 | 2.11 | OTU 29 | Nocardioidaceae | 0.57 | 0.57 | 0.53 |
| OTU 31 | Acidobacteria-6 iii1-15 | 1.26 | 1.11 | 0.96 | OTU 3 | CandidatusXiphinematobacter | 1.15 | 0.85 | 1.35 |
| OTU 33 | Pseudonocardia | 0.7 | 0.65 | 0.45 | OTU 30 | Actinobacteria MB-A2-108 | 0.63 | 0.54 | 0.56 |
| OTU 34 | Kaistobacter | 2 | 0.98 | 1.31 | OTU 308 | Nostocaceae | 2.37 | 2.25 | 2.43 |
| OTU 346 | Solirubrobacteraceae | 0.57 | 0.95 | 0.72 | OTU 31 | Acidobacteria-6 iii1-15 | 0.92 | 1.04 | 1.39 |
| OTU 35 | Anaerolineae | 1.37 | 2.06 | 1.13 | OTU 33 | Pseudonocardia | 0.62 | 0.5 | 0.51 |
| OTU 38 | Acidobacteria-6 iii1-15 | 1.18 | 1.01 | 0.88 | OTU 330 | Caldilineaceae | 0.92 | 0.62 | 0.86 |
| OTU 39* | Steroidobacter | 0.92 | 0.97 | 0.86 | OTU 34 | Kaistobacter | 1.69 | 1.25 | 1 |
| OTU 40 | [Pedosphaerales] | 1.19 | 0.97 | 1.07 | OTU 340 | Anaerolinea | 0.71 | 1.43 | 0.71 |
| OTU 41 | Anaerolinea | 1.56 | 1.18 | 1.04 | OTU 346 | Solirubrobacteraceae | 0.79 | 0.72 | 0.7 |
| OTU 42 | Gaiellaceae | 0.97 | 0.96 | 0.85 | OTU 349* | Ramlibacter | 2.28 | 1.23 | 1.47 |
| OTU 44 | [Pedosphaerales] auto67_4W | 1.19 | 1.15 | 1.42 | OTU 35 | Anaerolineae | 0.66 | 0.83 | 0.88 |
| OTU 48 | Bacillus longiquaesitum | 0.98 | 1.21 | 0.88 | OTU 36 | Rhodospirillaceae | 0.61 | 0.58 | 0.55 |
| OTU 488 | Chloroflexi Ellin6529 | 0.64 | 0.66 | 0.49 | OTU 38 | Acidobacteria-6 iii1-15 | 0.83 | 0.98 | 1.1 |
| OTU 49 | Syntrophobacteraceae | 0.61 | 0.92 | 0.6 | OTU 39* | Steroidobacter | 0.44 | 0.42 | 0.64 |
| OTU 492 | [Chthoniobacteraceae] | 1.03 | 0.95 | 1.11 | OTU 40 | [Pedosphaerales] | 1.24 | 0.8 | 1.03 |
| OTU 5 | Bradyrhizobium elkanii | 0.51 | 0.54 | 0.68 | OTU 41 | Anaerolinea | 0.72 | 1.75 | 0.79 |
| OTU 50 | Acidobacteria-6 iii1-15 | 0.74 | 0.79 | 0.82 | OTU 42 | Gaiellaceae | 0.48 | 0.53 | 0.6 |
| OTU 51 | Phormidium | 2.52 | 2.74 | 3.48 | OTU 44 | [Pedosphaerales] auto67_4W | 1.57 | 1.08 | 1.18 |
| OTU 52 | Clostridium bowmanii | 1.04 | 0.9 | 1.47 | OTU 48 | Bacillus longiquaesitum | 0.94 | 1.05 | 1.43 |
| OTU 53 | Chloroflexi Ellin6529 | 0.62 | 0.68 | 0.76 | OTU 488 | Chloroflexi Ellin6529 | 0.98 | 0.93 | 0.81 |
| OTU 54 | Anaerolineae envOPS12 | 0.92 | 1.17 | 2.72 | OTU 49 | Syntrophobacteraceae | 0.45 | 0.47 | 0.4 |
| OTU 55 | [Chloracidobacteria] RB41 | 0.74 | 0.54 | 1 | OTU 5 | Bradyrhizobium elkanii | 0.78 | 0.83 | 0.77 |
| OTU 56 | Rhodoplanes | 0.73 | 1.24 | 1.23 | OTU 50 | Acidobacteria-6 iii1-15 | 1.01 | 0.99 | 0.84 |
| OTU 57 | Chloroflexi S085 | 0.66 | 0.85 | 0.66 | OTU 51 | Phormidium | 2.61 | 3.73 | 3.65 |
| OTU 58 | Acidobacteria-6 iii1-15 | 1.18 | 0.77 | 1.09 | OTU 53 | Chloroflexi Ellin6529 | 0.86 | 1.06 | 1.04 |
| OTU 59 | Anaeromyxobacter | 0.7 | 0.47 | 0.54 | OTU 54 | Anaerolineae envOPS12 | 1.23 | 0.93 | 0.94 |
| OTU 6 | Balneimonas | 0.94 | 0.81 | 0.93 | OTU 55 | [Chloracidobacteria] RB41 | 0.63 | 0.68 | 0.78 |
| OTU 60 | Acidimicrobiales C111 | 1.13 | 0.85 | 1.1 | OTU 56 | Rhodoplanes | 1 | 0.89 | 0.94 |
| OTU 61 | Alcaligenaceae | 0.77 | 0.76 | 0.6 | OTU 57 | Chloroflexi S085 | 0.66 | 0.52 | 0.46 |
| OTU 65 | Piscirickettsiaceae | 1 | 1.03 | 1.1 | OTU 58 | Acidobacteria-6 iii1-15 | 1.44 | 0.97 | 1.15 |
| OTU 67 | Rhodospirillales | 1.17 | 0.78 | 1.01 | OTU 6 | Balneimonas | 1.28 | 1.44 | 1.14 |
| OTU 69 | Acidobacteria-6 iii1-15 | 0.83 | 0.74 | 0.8 | OTU 60 | Acidimicrobiales C111 | 0.95 | 0.78 | 1.07 |
| OTU 7 | Knoellia subterranea | 1 | 1.46 | 1.44 | OTU 61 | Alcaligenaceae | 0.84 | 0.62 | 0.63 |
| OTU 71* | Pseudomonas umsongensis | 5.41 | 4.4 | 4.35 | OTU 67 | Rhodospirillales | 1.34 | 0.9 | 0.92 |
| OTU 72 | Anaerolineae envOPS12 | 0.91 | 1.28 | 0.99 | OTU 69 | Acidobacteria-6 iii1-15 | 0.62 | 0.73 | 0.64 |
| OTU 74 | Bacillaceae | 1.06 | 1.42 | 1.15 | OTU 7 | Knoellia subterranea | 1.39 | 1.03 | 1.14 |
| OTU 740 | Verrucomicrobiaceae | 1.3 | 1.49 | 0.89 | OTU 71* | Pseudomonas umsongensis | 3.22 | 2.36 | 2.59 |
| OTU 744* | Acidimicrobiales C111 | 0.93 | 1.06 | 1.33 | OTU 72 | Anaerolineae envOPS12 | 0.62 | 1.29 | 1.07 |
| OTU 76 | Gaiellaceae | 1.14 | 1.08 | 1.3 | OTU 744* | Acidimicrobiales C111 | 0.76 | 0.8 | 0.76 |
| OTU 7805 | Chloroflexi Gitt-GS-136 | 0.52 | 0.68 | 0.63 | OTU 7981 | Gaiellaceae | 0.76 | 0.97 | 0.71 |
| OTU 7981 | Gaiellaceae | 0.72 | 0.64 | 0.71 | OTU 8 | Chloroflexi Ellin6529 | 1.07 | 0.81 | 0.94 |
| OTU 8 | Chloroflexi Ellin6529 | 0.69 | 0.43 | 0.52 | OTU 80 | Solirubrobacterales | 0.78 | 0.61 | 0.6 |
| OTU 80 | Solirubrobacterales | 0.65 | 1.12 | 1.01 | OTU 81 | Bacillus muralis | 0.97 | 0.79 | 0.93 |
| OTU 81 | Bacillus muralis | 1.28 | 1.41 | 1.28 | OTU 87 | Sinobacteraceae | 0.62 | 0.81 | 0.63 |
| OTU 88 | Phycisphaerae WD2101 | 0.52 | 0.58 | 0.79 | OTU 88 | Phycisphaerae WD2101 | 0.68 | 0.55 | 0.63 |
| OTU 8988* | Chloroflexi Ellin6529 | 0.89 | 0.61 | 1.14 | OTU 8988* | Chloroflexi Ellin6529 | 0.96 | 1.37 | 1.55 |
| OTU 9 | Anaerolinea | 1.96 | 1.74 | 1.31 | OTU 9 | Anaerolinea | 1.45 | 2.2 | 1.85 |
| OTU 92* | Rhodospirillales | 0.58 | 0.4 | 0.52 | OTU 902 | Anaerolinea | 0.73 | 1.49 | 0.78 |
| OTU 934 | Chloroflexi Ellin6529 | 0.71 | 0.8 | 0.78 | OTU 92* | Rhodospirillales | 0.74 | 0.7 | 0.61 |
| OTU 94 | Acidobacteria-6 iii1-15 | 0.86 | 1.05 | 1.15 | OTU 934 | Chloroflexi Ellin6529 | 0.86 | 1.05 | 0.9 |
| OTU 96 | Acidobacteria-6 iii1-15 | 0.59 | 0.34 | 0.4 | OTU 94 | Acidobacteria-6 iii1-15 | 1.42 | 1.49 | 1.14 |
| OTU 97 | Rhodoplanes | 0.62 | 0.76 | 0.57 | OTU 97 | Rhodoplanes | 0.76 | 0.67 | 0.63 |
| OTU 99 | Bacillus fumarioli | 0.92 | 1.2 | 0.88 | OTU 99 | Bacillus fumarioli | 0.66 | 0.44 | 0.6 |

*… OTUs with significant overall PERMANOVA of relative sequence abundance among treatments

Table S6: Effects of treatments, distance across the long side of the field, time and interactions on fungal and prokaryotic communities in soil of the field experiment based on BC dissimilarities assessed with overall PERMANOVA and corrected p-value of pairwise PERMANOVA tests among untreated pots and treatments of the fungal communities.

| Test | Organism | Factor | Degrees of freedom | Sums of squares | Mean of sums of squares | Pseudo F-statistic | R2 | P-value |
| --- | --- | --- | --- | --- | --- | --- | --- | --- |
| Overall | Fungi | Treatment | 4 | 0.6326 | 0.1581 | 1.4070 | 0.0557 | 0.0029 |
|  |  | Distance | 1 | 0.7987 | 0.7987 | 7.1062 | 0.0704 | 0.0001 |
|  |  | Time | 2 | 0.9593 | 0.4796 | 4.2672 | 0.0845 | 0.0001 |
|  |  | Treatment x time | 8 | 0.7677 | 0.0960 | 0.8538 | 0.0676 | 0.9681 |
|  |  | Distance x time | 2 | 0.2100 | 0.1050 | 0.9344 | 0.0185 | 0.6174 |
|  |  | Residuals | 71 | 7.9804 | 0.1124 | NA | 0.7032 | NA |
|  |  | Total | 88 | 11.3487 | NA | NA | 1.0000 | NA |
|  | Prokaryota | Treatment | 4 | 0.3122 | 0.0781 | 1.0012 | 0.0438 | 0.4105 |
|  |  | Distance | 1 | 0.2655 | 0.2655 | 3.4055 | 0.0373 | 0.0014 |
|  |  | Time | 2 | 0.4300 | 0.2150 | 2.7578 | 0.0604 | 0.0013 |
|  |  | Treatment x time | 8 | 0.3936 | 0.0492 | 0.6311 | 0.0553 | 1.0000 |
|  |  | Distance x time | 2 | 0.1059 | 0.0529 | 0.6791 | 0.0149 | 0.9664 |
|  |  | Residuals | 72 | 5.6133 | 0.0780 | NA | 0.7883 | NA |
|  |  | Total | 89 | 7.1206 | NA | NA | 1.0000 | NA |
|  |  |  |  |  |  |  |  |  |
|  |  | Pairwise comparison of each treatment with untreated (p-value) | | | |  |  |  |
|  |  | F_cap_ | FCBK | F_gran_ | Insec |  |  |  |
| Pairwise | Fungi | 0.522 | 0.522 | 0.124 | 0.522 |  |  |  |
