# Supplementary figures and images for: Assessing effects of the entomopathogenic fungus Metarhizium brunneum on soil microbial communities in Agriotes spp. biological pest control
Source: FEMS Microbiol Ecol. 2017 Sep 11;93(10):fix117. doi: 10.1093/femsec/fix117 (PMC5812499; doi:10.1093/femsec/fix117)

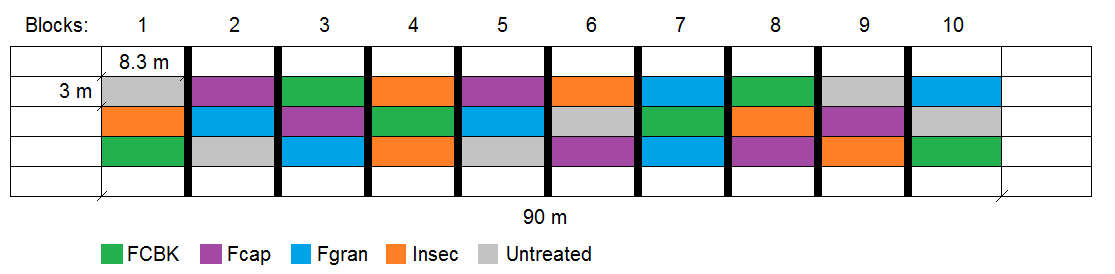

Supplement: Supplement Files [file fix117_supplement_files.zip › Supp_figure_1.png]

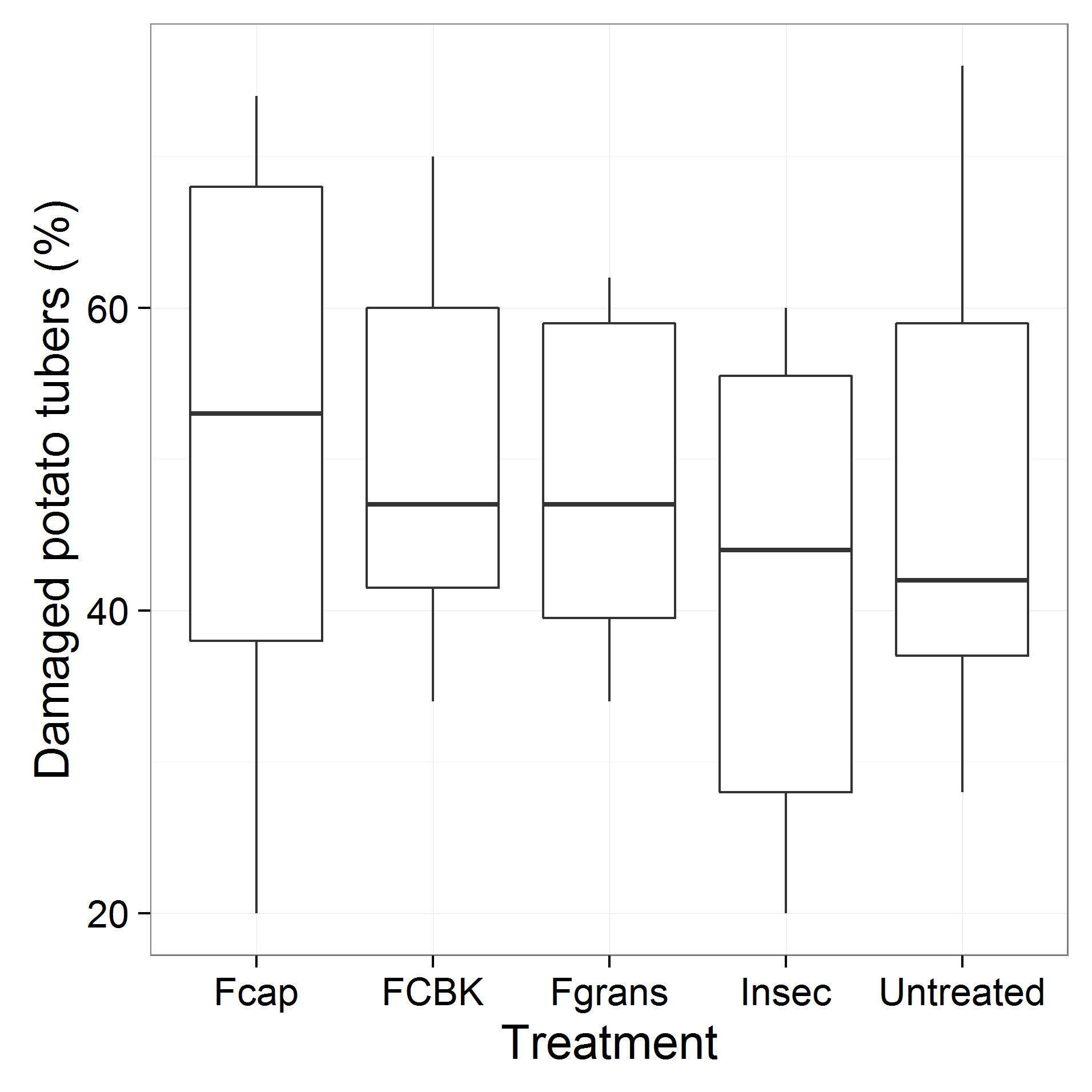

Supplement: Supplement Files [file fix117_supplement_files.zip › Supp_figure_2.tif]

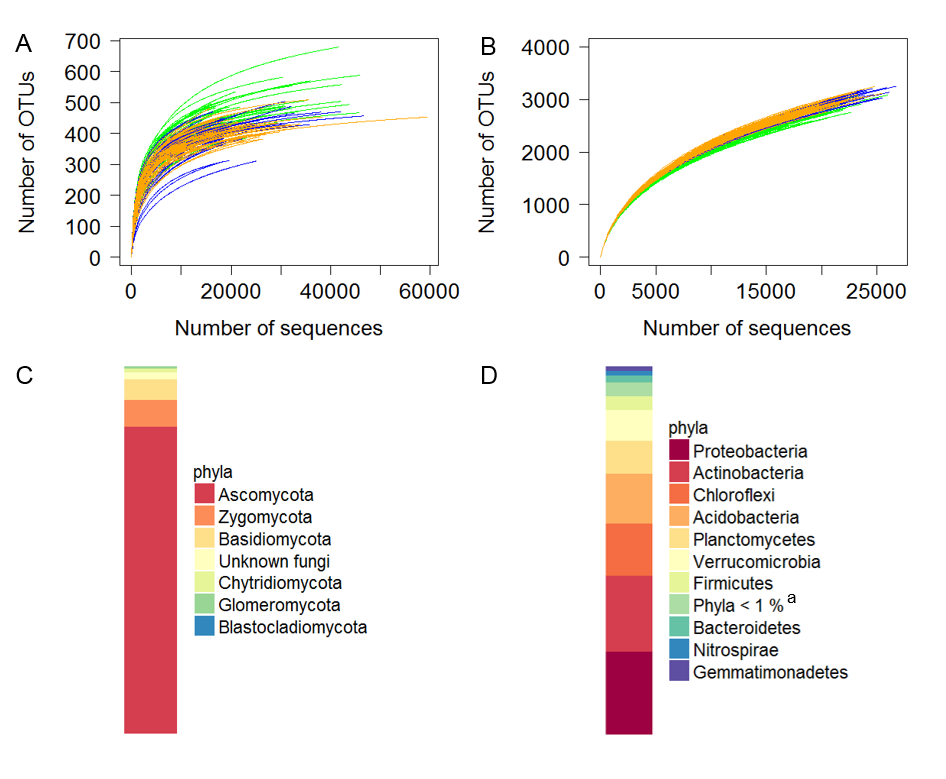

Supplement: Supplement Files [file fix117_supplement_files.zip › Supp_figure_3.tif]

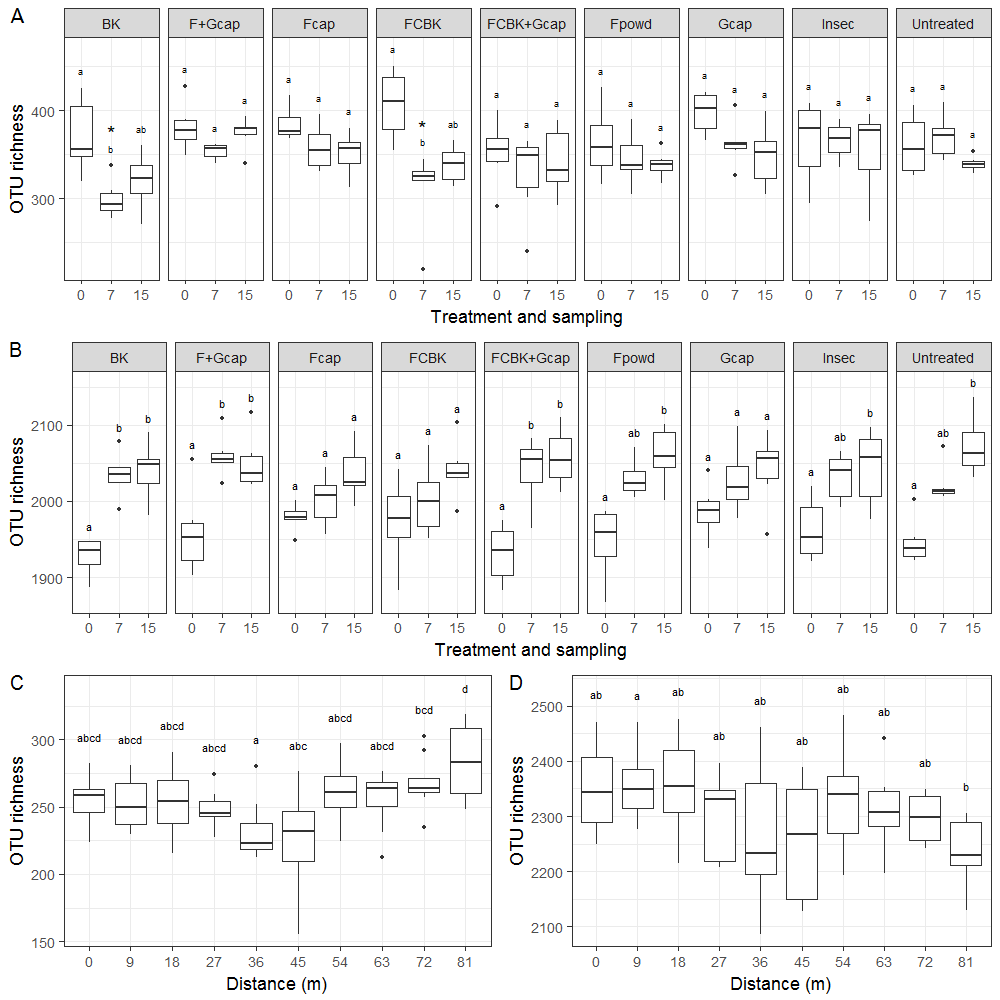

Supplement: Supplement Files [file fix117_supplement_files.zip › Supp_figure_4.png]

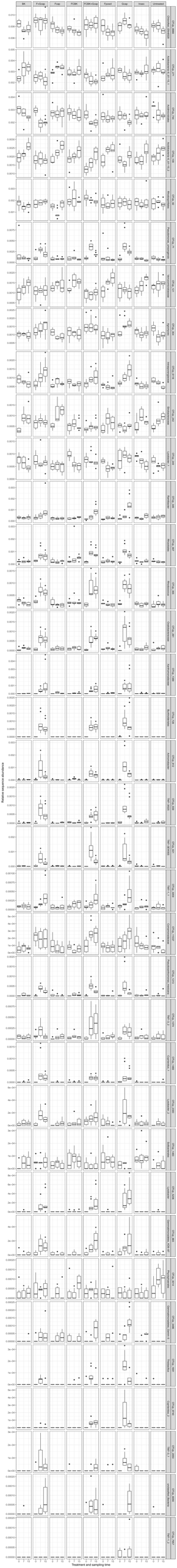

Supplement: Supplement Files [file fix117_supplement_files.zip › Supp_figure_5.pdf]

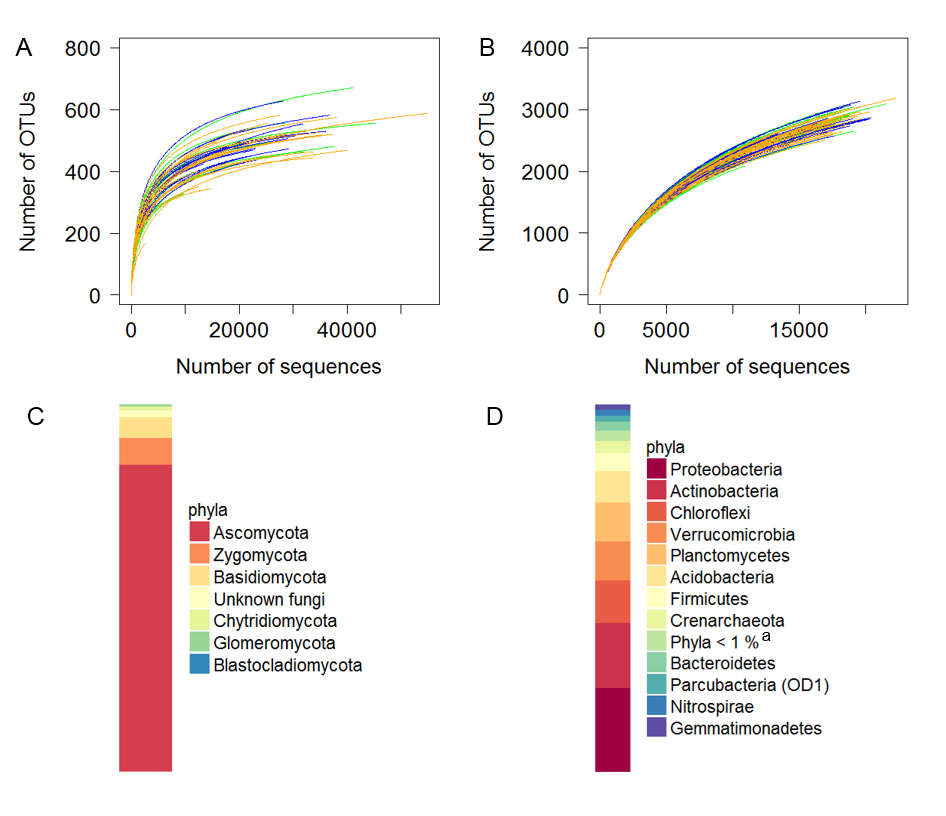

Supplement: Supplement Files [file fix117_supplement_files.zip › Supp_figure_6.tif]
